# Glycoproteins gM and gN are indispensable factors for rhesus macaque rhadinovirus replication and spread but can be reconstituted by KSHV chimeras

**DOI:** 10.1128/jvi.01922-24

**Published:** 2025-02-25

**Authors:** Gavin Golas, Byung S. Park, Scott W. Wong

**Affiliations:** 1 Vaccine and Gene Therapy Institute, Oregon Health & Science University 6684 , Beaverton, Oregon, USA; 2 Biostatistics Shared Resource, Oregon Health & Science University, Knight Cancer Institute 608031 , Portland, Oregon, USA; 3 Biostatistics and Bioinformatics Core, Oregon National Primate Research Center, Beaverton, Oregon, USA; 4 Division of Pathobiology and Immunology, Oregon National Primate Research Center, Beaverton, Oregon, USA; 5 Department of Molecular Microbiology and Immunology, Oregon Health & Science University 547642 , Portland, Oregon, USA; Lerner Research Institute, Cleveland Clinic, Cleveland, Ohio, USA

**Keywords:** Kaposi's sarcoma-associated herpesvirus, rhesus macaque rhadinovirus, primate rhadinovirus, animal models, glycoproteins, nonsense mutations, chimeras, gM, gN, vaccine development

## Abstract

Rhesus macaque rhadinovirus (RRV) is a primate gamma-2 herpesvirus (rhadinovirus) closely related to Kaposi sarcoma-associated herpesvirus (KSHV), the human oncovirus that causes Kaposi sarcoma. Like other herpesviruses, KSHV and RRV encode numerous envelope glycoproteins involved in cell attachment, entry, as well as assembly and release of progeny virions from infected cells. Two glycoproteins postulated to form a complex and reported to be virus-neutralizing targets are glycoproteins M (gM) and N (gN). To investigate gM and gN in rhadinovirus infection, we utilized infectious and pathogenic bacterial artificial chromosomes (BAC). RRV BACmids with nonsense mutations introduced into gM or gN did not yield an infectious virus. However, when gM or gN of RRV were exchanged for gM or gN from KSHV, each of the KSHV-chimeric RRV BACmids restored virus replication and infectious spread. Interestingly, we also discovered that the substitution of KSHVgM into the RRV BACmid was associated with attenuation in viral spread, an effect that was not countered by a double-chimeric virus. In contrast, the substitution of RRV gN into a KSHV BACmid negatively affected the assembly of KSHV, independent of gM/gN complex formation. Therefore, here, we revealed that in KSHV and RRV, gM and gN are interchangeable, contribute to crucial functions for viral assembly and spread, and have evolved in a virus-specific manner. Although more research is needed to define the roles of gM and gN, our work establishes the first glycoprotein-chimeric viruses for KSHV and RRV, which can now be used to corroborate gM/gN as targets for a cancer vaccine.

**IMPORTANCE:**

Kaposi sarcoma (KS) is a human cancer caused by KSHV and is one of the most frequently occurring cancers in HIV/AIDS patients, as well as in regions where KSHV is endemic. In this report, we have constructed and authenticated the first KSHV glycoprotein-encoding chimeric viruses for evaluations in the RRV/rhesus macaque model and have also uncovered fundamental roles for the glycoproteins gM and gN. Our work is significant by successfully bridging the human-specific, species barrier that has previously restricted preclinical evaluations of the KSHV glycoproteins as vaccine targets *in vivo*. Although there is no KSHV-specific animal model that is widely used, these KSHV-chimeric viruses may be useful as tools to guide future vaccine design and strategy as vaccine candidates progress toward clinical trials.

## INTRODUCTION

Kaposi sarcoma-associated herpesvirus (KSHV), also known as human herpesvirus 8, is one of the internationally recognized, oncogenic viruses afflicting humans and is the etiological agent responsible for Kaposi sarcoma (KS), as well as B-cell lymphoproliferative disorders, such as primary effusion lymphoma and a variant of multicentric Castleman disease (1–4). Although most people infected by KSHV remain asymptomatic, the virus still inflicts a large disease burden, globally. Each year, there are approximately 35,000 new cases of KSHV-associated malignancy (2020) (5). Many of these cases occur in people living with HIV (PLWH), where the risk of developing KS is inversely correlated with CD4 count, consistent with other diseases of opportunistic pathogens. Accordingly, with the deployment of antiretroviral therapy (ART), the cumulative risk of developing HIV-associated cancers, including cancers caused by KSHV, has been substantially reduced (6).

However, despite this progress, the maladies linked to KSHV have not been eliminated, and the risks persist for millions of people living in areas where KSHV is endemic. Although ART will continue to endure as a cornerstone of prevention and treatment for HIV infection, not all PLWH who develop KS benefit from ART and not all KS patients have HIV. For PLWH, sometimes, instead of disease suppression, the initiation of ART can unpredictably cause disease acceleration (7). When a patient with KS does not respond to ART, including HIV-negative individuals, more intensive therapies such as systemic chemotherapy are often required. Furthermore, because the risk of developing KS increases with age (7), as PLWH—a group of ~40 million people—continue to live longer, the individual hazards associated with aging and HIV may become compounded in the future. For these reasons, the development of a KSHV-specific therapy or a vaccine would be highly beneficial, as a vaccine would be the most cost-effective measure to protect against, and ideally to prevent, KSHV-driven cancers.

However, the development of a KSHV vaccine faces unique challenges, which were recently reviewed (8). One of the most challenging barriers is a deficiency of animal models, due to the highly restricted tropism of KSHV (9). Although marmosets (10) and tree shrews (11) can be experimentally infected by KSHV, to some extent, this limited tropism still presents a major challenge for vaccine development.

Despite the absence of a KSHV-specific animal model that is universally accepted, there is an established nonhuman primate (NHP) model that relies on a simian homolog, which recapitulates important aspects of KSHV pathogenesis. Rhesus macaque rhadinovirus (RRV) is a gamma-2 herpesvirus that is highly related to KSHV (12, 13)—as their genomes are colinear, and individual genes are well conserved (14, 15). A pathogenic strain of RRV (17577) has been isolated that can cause B-cell lymphoproliferative disorders, lymphomas, and, in rare cases, a mesenchymal proliferative lesion (retroperitoneal fibromatosis) that possesses cellular features that resemble KS, in simian immunodeficiency virus (SIV)-infected rhesus macaques (RM) (12, 16). This disease model in NHPs closely parallels KSHV/HIV coinfection in humans and is a valuable asset to advance our understanding of primate rhadinovirus infection and pathogenesis *in vivo* (17).

As viral glycoproteins are conventional vaccine targets for most viruses, they could also be vaccine targets for KSHV. To investigate which KSHV glycoproteins might be the strongest neutralization targets of natural humoral immunity, in one study, plasma derived from KSHV-infected individuals was absorbed by cells expressing individual KSHV glycoproteins (18). Although all glycoproteins studied were found to have some degree of neutralization potential, a few stood out, including gH and gL (recognized targets of virus neutralization in other herpesviruses), as well as gM and gN. Although antibodies recognizing gM and/or gN might be less frequent in the blood of KSHV-infected individuals, they were found to strongly neutralize KSHV, raising the possibility that these proteins might be unexplored targets of KSHV neutralization.

Glycoproteins gM and gN are widely conserved among animal herpesviruses (19) and are encoded by all known members of the human herpesviruses. This distinction is actually quite rare among herpesvirus proteins, with only a handful of representatives (20), considering the evolutionary gap between the alpha-, beta-, and gamma-herpesvirus subfamilies is thought to span hundreds of millions of years (19, 21). In contrast to the well-known glycoproteins in herpesviruses gB, gH, and gL, far less is understood for gM and gN. In human cytomegalovirus (HCMV), gM is actually the most abundant envelope protein by mass spectrometry (22), signifying a major presence and likely a major contribution to the virus (23). Typically, gM and gN are often studied together, since they can form a disulfide-bonded protein complex (24–29). So far, the gM/gN heterodimer appears to be universal for all known human herpesviruses. Importantly, the gM/gN complex has also been shown to be a promising target of virus-neutralizing antibodies (Abs) for HCMV (30–32). Co-immunization with gM and gN vaccines was able to induce neutralizing Abs, which correlated with the ability to protect mice against a lethal challenge by murine CMV (33). Whether these vulnerabilities and opportunities for therapeutics also extend to gammaherpesviruses (such as KSHV) is currently unclear. Despite broad conservation among herpesviruses, the roles of gM and gN from each herpesvirus should be independently assessed, so that their requirement for virus replication and spread, and potential as vaccine targets, can be individually delineated.

In 2003, Koyano et al*.* performed the initial (and only prior) characterization of KSHV gM and gN. In this paper, they concisely showed that KSHV gM and gN are both N-glycosylated, appear to form a complex, and may regulate viral fusion (28). Later, mass spectrometry studies confirmed that gM and gN are indeed incorporated into mature KSHV virions (34, 35). However, the specific roles of gM and gN for KSHV are unknown, and their homologs in RRV have not yet been investigated.

KSHV/RRV gM is encoded by ORF39 and predicted to encode a multi-pass transmembrane glycoprotein with eight transmembrane domains that comprise the body of the protein. This unique structure is expected to confer a highly hydrophobic protein, which may have enhanced stability in viral and/or cellular membranes. At the C-terminus, gM harbors a long, cytoplasmic tail (CT), which should be expressed intracellularly and inside the mature viral particle. On the other hand, KSHV/RRV gN is encoded by ORF53 and predicted to encode a small, single-pass transmembrane glycoprotein. Since gM and gN require further dissection in KSHV and RRV, both as individual proteins and as a complex, and because they may be vaccine targets, we decided to focus our study on them.

In this work, we developed novel gM- and gN-recombinant RRVs, as well as KSHV-chimeric RRVs that encode and express KSHV gM and gN in place of their RRV homologs. We used these viruses to support the conclusions that gM and gN are essential for RRV; however, the substitution of the corresponding KSHV glycoprotein can rescue the growth-impaired phenotypes of the viruses encoding gM- or gN-nonsense mutations. We also found that a KSHV/RRV chimera expressing KSHV gM in RRV was highly attenuated, whereas a KSHV/RRV chimera expressing a similar gM/gN complex in KSHV also led to abnormalities. Taken together, our work advances the understanding of gM and gN as necessary glycoproteins in the life cycle of the oncogenic gammaherpesvirus RRV, pushes the frontier in our understanding of gM and gN in KSHV and RRV, and provides new infection models to conduct KSHV vaccine-related studies *in vitro* and potentially *in vivo*.

## RESULTS

To gain a comprehensive comparison of gM and gN from KSHV and RRV, relative to all other viral glycoproteins, we first compared the protein homologies of all probable envelope proteins in KSHV with their homologs in RRV (Table 1). Here, we found that gM and gN were among the most highly conserved glycoproteins between KSHV and RRV. In fact, gM and gN had higher homologies than either gH or gL and were nearly as high as gB (which is often used as a marker of evolution in herpesviruses). Specifically, the KSHV/RRV gMs have 56% identity and 76% similarity, whereas the KSHV/RRV gNs have 48% identity and 63% similarity.

**TABLE 1:** Alignments for KSHV and RRV glycoproteins^*a*^

	Length (AA)		Identity	Similarity	Gaps	GAL (AA)	N-W score
	KSHV	RRV					
gB	845	829	64.3%	79.5%	04.2%	855	3705.5
gH	730	704	39.3%	61.3%	05.2%	736	1828.0
gL	167	169	27.5%	41.0%	11.2%	178	0251.0
gM	400	378	56.4%	76.3%	06.0%	401	1632.5
gN	110	104	47.7%	63.1%	07.2%	111	0328.0
K8.1/R8.1	228	275	19.4%	30.7%	42.3%	319	0151.0
ORF4	550	645	30.3%	44.2%	22.7%	674	1045.5
ORF27	290	269	27.3%	47.7%	16.1%	304	0422.0
ORF28	102	91	21.7%	31.0%	50.4%	129	0097.5
ORF58	357	360	39.4%	60.1%	06.7%	371	0852.5

^*a*^ Pairwise alignments of KSHV and RRV glycoproteins using EMBOSS, using the Needleman-Wunsch (N-W) algorithm. BLOSUM50 was utilized as a substitution matrix. Sequences were derived from BAC16/JSC-1 KSHV (GQ994935.1) and 17577 RRV (AF083501.3). GAL: Global Alignment Length.

**Fig 1:**
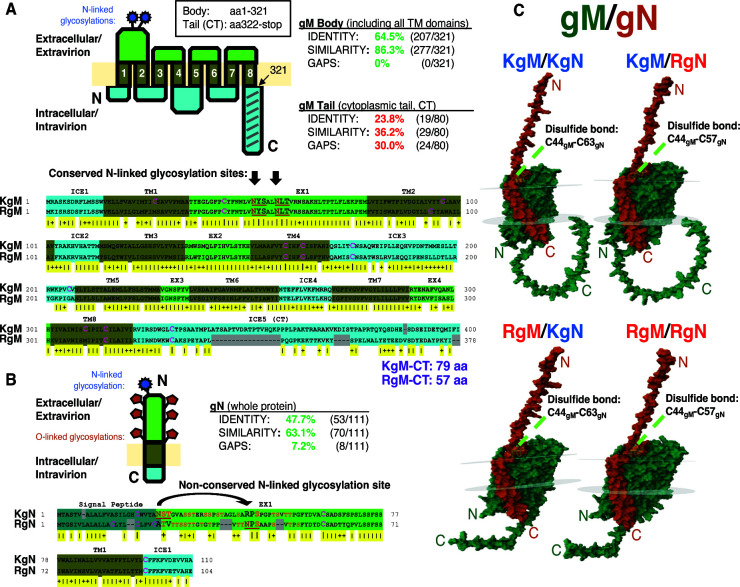
Structural comparisons of gM, gN, and the gM/gN complex from KSHV and RRV. (**A**) Protein alignments of KSHVgM (KgM) (ACY00437.1, from BAC16/JSC-1 KSHV, GQ994935.1) and RRVgM (RgM) (AAD21366.1, in 17577 RRV, AF083501.3), produced using EMBOSS, were color-coded by membrane topology, as determined by DeepTMHMM. To determine amino acid similarity, the BLOSUM50 matrix was applied. N-linked glycosylation motifs were identified and are underlined. Cysteines are marked in pink. (**B**) Similar analytic tools and markings were applied to compare KSHVgN (KgN) (ACY00452.1, in BAC16/JSC-1 KSHV) and RRVgN (RgN) (AAD21379.1, in 17577 RRV). Residues that were identified as probable O-glycosylation sites are marked in orange. (**C**) gM/gN complexes from KSHV, RRV, as well as KSHV/RRV crosses, were generated using AlphaFold and visualized with PDB-3D viewer (gM in green, gN in orange).

Next, we performed protein alignment and structural analyses on KSHV and RRV gM (KgM and RgM) (Fig. 1A). Both proteins were predicted to encode exceptionally similar structures. The gMs were both predicted to form eight transmembrane helices, forming four ectodomains and five intracellular domains. All ectodomains are highly conserved, including the first ectodomain (37/42 aa identical, 39/42 aa similar), which comprises the largest loop. The first ectodomain also encodes two N-linked glycosylation sites, which, in KSHV and RRV, are positionally conserved and are identical in sequence. Although the overall level of homology between KSHV and RRV gM is very high, this is greatly reduced at the fifth intracellular domain, referred to as the CT. Within the body of the KSHV and RRV gMs (aa1-321 for both proteins), there is 64.5% identity, 86% similarity, and no gaps. However, in the CTs, these values are diminished considerably, down to 24% identity, 36% similarity, and multiple gaps. The divergence of the CTs is also apparent by the length of the CTs, with the KgM-CT (79 aa) being 22 aa longer than the RgM-CT (57 aa), accounting for the overall size difference between KgM (400 aa) and RgM (378 aa). These structural analyses indicate that although there is extensive conservation between the two gMs, there are also a few prominent differences, located mainly in the CT.

Similarly, we conducted structural analyses on KSHV and RRV gN (KgN and RgN) (Fig. 1B). Both gNs were predicted to encode a small, single-pass transmembrane glycoprotein that is ~100 aa (KgN: 110 aa, RgN: 104 aa), as in other herpesviruses, with a short, 12-aa CT that is highly conserved between the two viruses. Predictions for N-linked glycosylation sites identified that each gN may have one site—but, they are neither positionally conserved nor are they conserved in identity. Additionally, we found that both gNs have many potential O-glycosylation sites, with ~25% of the residues in each gN ectodomain serving as viable sites.

To screen the ability of gM and gN to form heterodimeric complexes *in silico*, we used Alphafold (Fig. 1C) (36, 37). Alphafold predicted gM/gN complexes with high confidence, although pLDDT scores (confidence per residue) were slightly lower at the N-terminus for the gNs and much lower at the CT for the gMs. Still, the gM/gN complexes were modeled with extraordinary consistency, even for the gM/gN crosses (pairings with a different virus). In all representations of the gM/gN complex, potential bonding was observed between the ectodomain of gN with the first and second ectodomains of gM, whereas the membrane domain of gN interacted with the first and third transmembrane domains of gM. A disulfide bond was also identified between C44 of gM and C63 of KgN (corresponding to C57 of RgN), similar to what has been described for other gM/gN complexes (27). This disulfide bond may help stabilize the complex, but noncovalent interactions, such as hydrophobic forces, are also likely important. Considering these comparisons and potential structures, gM and gN of KSHV and RRV are highly conserved and suggest that gM/gN complexes may form via conserved interactions. Therefore, we rationalized that it might be possible to develop gM− and gN− chimeric viruses.

### Development of gM and gN nonsense RRVs

**Fig 2:**
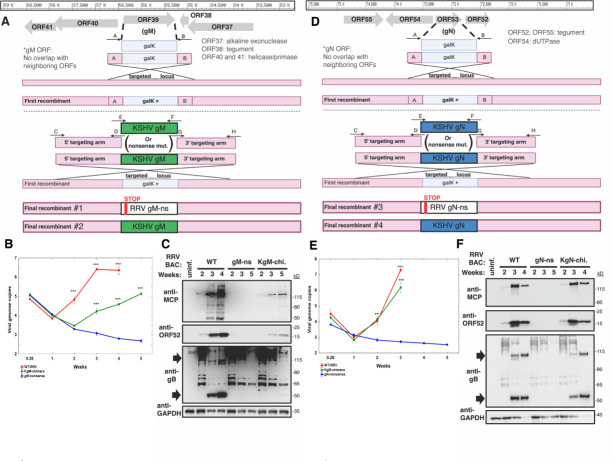
gM and gN are required for RRV spread, whereas replacement with KSHV gM or gN resulted in replication-competent virus. (**A**) Schematic of BACmid mutagenesis at the RRV gM locus. (**B, C**) qPCR of BAC-transfected 1°RFs, using DNA isolated from cells, presented as viral genome copies log10 quantity(B). (**C**) Western blot using anti-RRV antibodies, recognizing RRV-infected cells. (**D**) Schematic of BACmid mutagenesis at the RRV gN locus. (**E, F**) qPCR of BAC-transfected 1°RFs, using DNA isolated from cells, presented as viral genome copies log10 quantity (**E**). (**F**) Western blot using anti-RRV antibodies, recognizing RRV-infected cells. The black arrows point to perceived mature forms of gB. Viral genome replication was analyzed as described in the Material and Methods. *P* values of less than 0.0001 (***) were considered extremely significant, whereas *P* values of less than 0.005 (**) were considered significant.

Since gM and gN have not been studied previously in RRV, it was unknown what the effect would be of removing these endogenous glycoproteins. Hence, we generated gM− and gN− nonsense (ns) BAC clones, by inserting 5’ stop codons into RRV gM and gN, utilizing two-step homologous recombination (Fig. 2A and D)—by first replacing the RRV gM or gN open reading frame (ORF) with a selectable insert (galK), and then repairing that insert with DNA encoding ns mutations.

Once the recombinant gM BAC clones were verified by PCR/Sanger sequencing and further validated by restriction digest and Southern blot (Fig. A1), we transfected the recombinant BAC clones into primary rhesus fibroblasts (1^o^RF), which support robust lytic infection of RRV, to test whether viruses could be reconstituted. We surveyed for the development of viral cytopathic effect (CPE) over the course of several weeks. In comparison to wild-type (WT) RRV BAC, which easily formed CPE, no CPE was detected for the gM-ns RRV BAC in five rounds of transfection. To measure these observations more quantitatively, we performed qPCR on the BAC-transfected cells to measure viral genome replication (Fig. 2B). In agreement with the absence of CPE, we found the gM-ns RRV BAC-transfected cells did not support viral genome replication. Likewise, at the protein level, gM-ns RRV BAC expressed canonical RRV antigens (MCP, ORF52, and gB) to a much lower level than with WT RRV BAC (Fig. 2C). Interestingly, we did observe some gB signals for gM-ns RRV BAC by western blot, as well as for the other RRV BACs. However, we think that these signals may represent immature forms of gB, as the dominant form of gB associated with mature RRV particles has been reported to be close to 55 kD (38), and in our hands at 55 kD and ~110 kD. Consistent with this idea, these potential immature forms of gB decreased as the 55 kD and ~110 kD bands increased for WT RRV BAC (Fig. 2C and F). We also note that these potential immature forms of gB may be more readily detected following RRV BAC transfection compared with primary infection (unpublished observations). Nevertheless, these data suggested that although the gM-ns RRV BAC had been successfully transfected, it was unable to replicate and spread.

Next, to assess the role of gN in infectious spread, after gN-ns RRV BAC was constructed by homologous recombination and confirmed by PCR/Sanger sequencing, as well as by restriction digestion and Southern blot (Fig. A2), the recombinant BAC was transfected into 1^o^RFs to try to reconstitute infectious virus. However, there was no virus-associated CPE observed for the gN-ns RRV BAC by 1 month post-transfection, on two separate attempts (10 wells total). In qPCR (Fig. 2E) and western blot (Fig. 2F) analyses, there was also no sign of virus replication for the gN-ns RRV BAC in transfected cells. As with gM-ns RRV BAC-transfected cells, we again detected potentially immature forms of gB by western blot, suggesting that the gN-ns RRV BAC had been successfully transfected into cells. These data revealed that both gM and gN may be required for RRV to effectively disseminate *in vitro*.

### Development of recombinant RRV chimeras with KSHV gM and gN

To test the abilities of KSHV gM and gN to function in place of RRV gM and gN, we created recombinant RRV chimeras encoding full-length KSHV gM or gN. For our models, we used KSHV sequences derived from a prototype KSHV (JSC-1/BAC16). Fortunately, our approach benefitted from the fact that neither gM nor gN ORFs overlapped with known viral ORFs in RRV. The opposite is also true for KSHV; hence, no partial ORFs would have been carried over as byproducts into the chimeras. In our designs, each KSHV ORF completely replaced each RRV ORF—exactly from the start codon to the stop codon.

To create the chimeric RRVs, we also used homologous recombination (Fig. 2A and D), and after validating the BACmids by PCR/sequencing, as well as restriction digest and Southern blot (Fig. A1 and A2), we transfected 1^o^RFs with the KSHV-chimeric RRV BACmids. With KgM substituted at the gM locus, a modest amount of viral CPE was observed, which developed slowly and was visibly reduced compared with WT RRV BAC. Accordingly, KSHVgM-chimeric RRV (KgM-chi. RRV) BAC also displayed evidence of productive viral replication by qPCR, as indicated by an increase in the viral load by more than 300-fold in cells from 2 to 5 weeks post-transfection (Fig. 2B). However, these replication dynamics seemed to be delayed considerably compared with WT RRV BAC. In western blot, the KgM-chi. RRV BAC also expressed several markers of RRV infection to a modest level (Fig. 2C). On the other hand, with KgN substituted at the gN locus, KSHVgN-chimeric RRV (KgN-chi. RRV) BAC generated vigorous viral CPE and seemed to be as replication competent as WT RRV BAC (Fig. 2E). KgN-chi. RRV BAC strongly expressed the representative panel of RRV antigens (Fig. 2F). Collectively, these data showed that both KgM- and KgN- chimeric RRV BACmids were competent for viral replication and spread, unlike the gM- and gN- ns BACmids, which were clearly defective. These data also suggested that RRV may have tolerated the substitution with KgN better than KgM.

### Expression of gM and gN in KSHV-chimeric viruses

**Fig 3:**
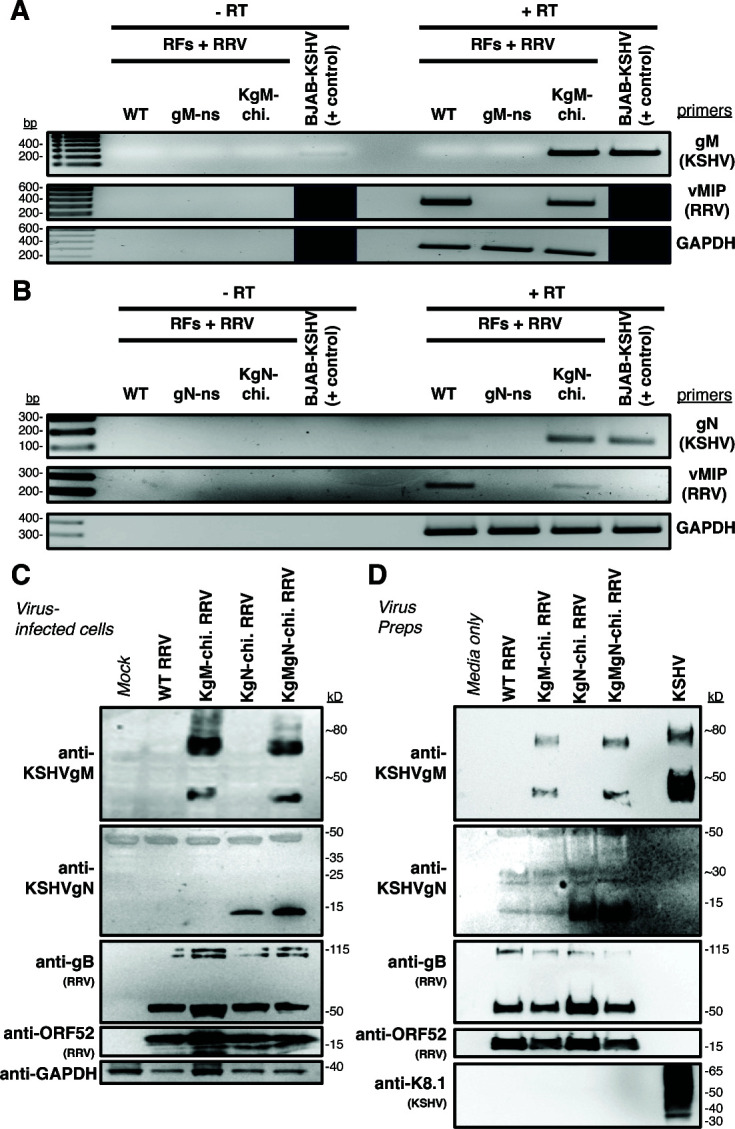
Glycoprotein-chimeric RRVs express KSHV gM and gN. (**A-B**) At 3–4 weeks post-BAC transfection, RT-PCR was used to examine the gene expression in infected cells, using primers specific for KSHVgM, KSHVgN, vMIP (as a marker for RRV RNA), or GAPDH (as a marker of total RNA). As negative controls, reverse transcriptase (RT) was excluded. As a positive control for the expression of the KSHV genes, RNA was isolated from lytically-reactivated KSHV+ cells (Brk219 cells). (**C**) After constructing, plaque purifying, and titering, single- and double-chimeric RRVs were used to infect 1°RFs (MOI: 1.0), and infected cell lysates were analyzed by western blot for the expression of the KSHV proteins, using newly generated, KSHVgM- and KSHVgN-specific Abs, as well as by RRV-specific Abs. (**D**) Gradient-purified viruses were also evaluated for the incorporation of KSHV proteins by western blot.

To see if the chimeric viruses expressed the KSHV glycoproteins, we initially used RT-PCR (Fig. 3A and B). Encouragingly, we found that KgM or KgN RNA transcripts were expressed in corresponding chimeric virus-infected cells. Building on these data, we next analyzed whether the chimeric viruses expressed the KSHV glycoproteins as proteins. Unfortunately, the original KSHV gM and gN rabbit antisera produced 20 years ago were no longer available. Thus, we immunized New Zealand white rabbits with peptides specific to either KSHV gM or gN and utilized the resulting polyclonal, affinity-purified Abs on chimeric virus-infected cell lysates (Fig. 3C) and confirmed that the chimeric viruses successfully expressed either KgM or KgN protein in the corresponding virus-infected cells. More importantly, western blot analysis on gradient-purified, chimeric virus preparations also detected KgM and KgN, demonstrating the ability of virus particles to incorporate KgM and KgN (Fig. 3D).

In addition to the two, single glycoprotein KSHV chimeras, we also combined these insertions to generate a double-chimeric virus (KgMgN-chi. RRV) BAC (Fig. A2), which simultaneously expressed both KSHV proteins (Fig. 3C and D). Therefore, we found that RRV can express gM and gN from KSHV, in place of its homologs.

To confirm the viral DNA sequences, we sequenced the viral genomes of the KSHV chimeras. This verified that KgM-chi. RRV (Genbank: PQ507925) and KgN-chi. RRV (Genbank: PQ507926) encoded the correct aa sequence of KgM or KgN. However, in KgMgN-chi. RRV, we detected a point mutation in KgM (nt.28C > A, 10L > I) (Genbank: PQ507927). This mutation had not been present in the source BACmid; hence, it arose naturally in the virus during passaging. In terms of structure, aa10 is near the start of the first transmembrane domain. To determine whether this exchange might be beneficial for RRV or if it was just a bystander mutation, we re-transfected 1^o^RFs with the KgMgN-chi. RRV BAC and generated four independent, gradient-purified virus stocks (each grown in culture for about 3 months). Upon sequencing these viruses, with a total of 1,621 reads at nt.28 of gM, no mutations were found that deviated from the wild-type KgM or KgN aa sequence (Genbank: PQ507928). We considered re-developing RRV isolates devoid of the BAC cassette, but this would have been time consuming and would not have safeguarded from the acquisition of the same mutation or other mutations in the viral genome. Therefore, we rationalized that the switch from leucine to isoleucine would have a minimal effect, since these aa have similar properties, and the mutation was located distal to the ectodomains, and then decided to characterize KgMgN-chi. RRV (10L > I) along with the other KSHV chimeras.

### Growth kinetics of KgM-, KgN-, and KgMgN-chi. viruses

**Fig 4:**
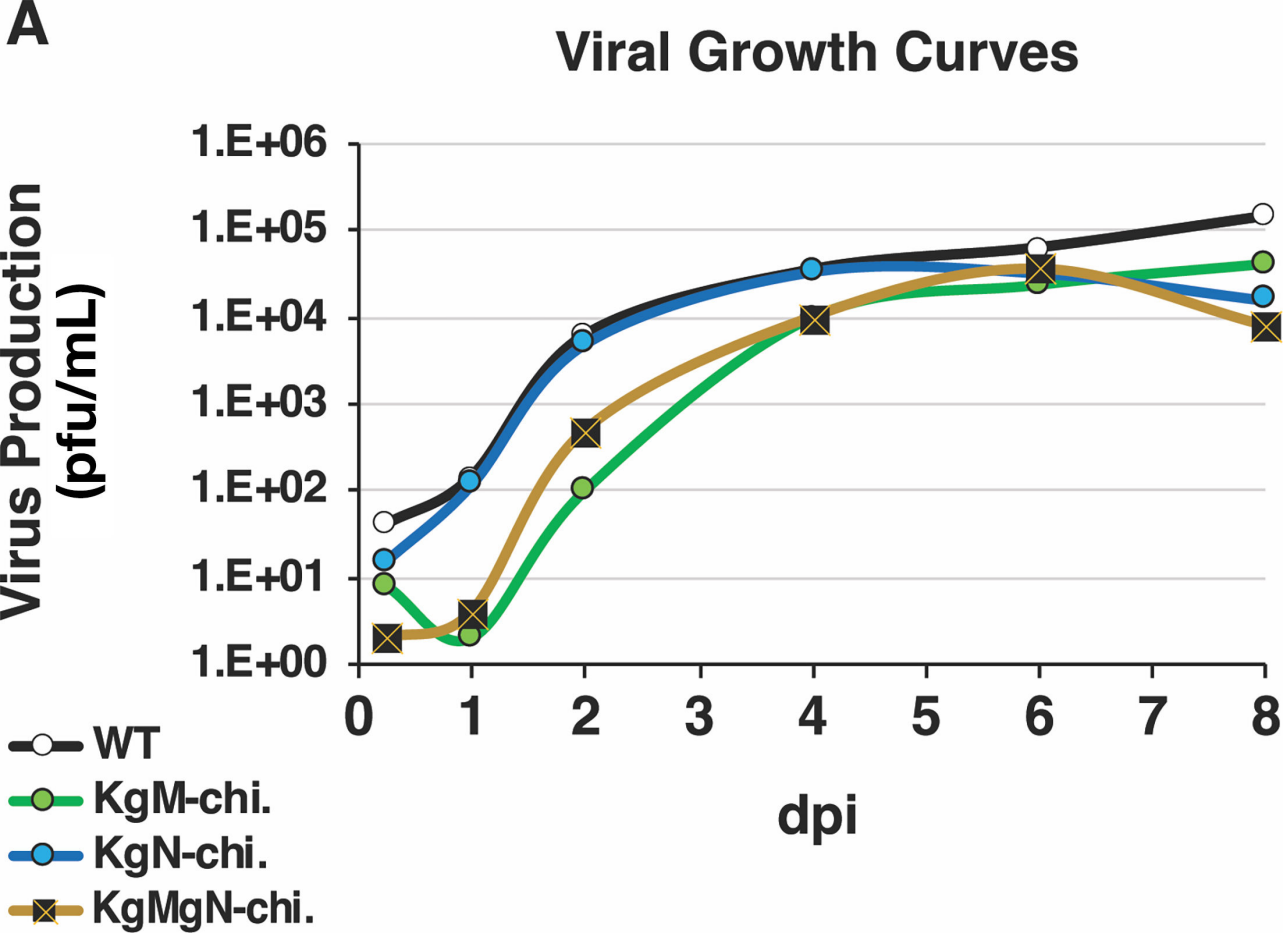
*In vitro* growth analyses of wild-type and KSHV-chimeric viruses. (**A**) Multi-step growth curve analysis was performed by infecting 1°RFs at an MOI of 0.1, using WT RRV BAC-derived virus as a control. At the time points shown, the cells and supernatants were harvested, freeze-thawed, and sonicated. Infectious virus was measured by plaque assay, with each time point assessment performed in duplicate.

To preliminarily characterize how the three KSHV chimeras grew compared with WT RRV, we infected 1^o^RFs for growth assays. In multi-step growth assays, virus production was highly efficient for WT RRV, as well as for KgN-chi. RRV, but it was strongly delayed for KgM-chi. RRV, which was not fully reversed by KgMgN-chi. RRV (Fig. 4A). Unfortunately, due to the extremely low yield of the KgM-chimeric RRV, we were not able to generate enough virus to test the viruses in a single-step growth assay. Nevertheless, with our earlier data, we concluded that the spread of KgM-chi. RRV is highly attenuated *in vitro*, and this phenotype persisted even when both proteins were encoded from KSHV. Overall, these data provided more evidence that the chimeric viruses had distinct growth properties.

### gM is essential for RRV

**Fig 5:**
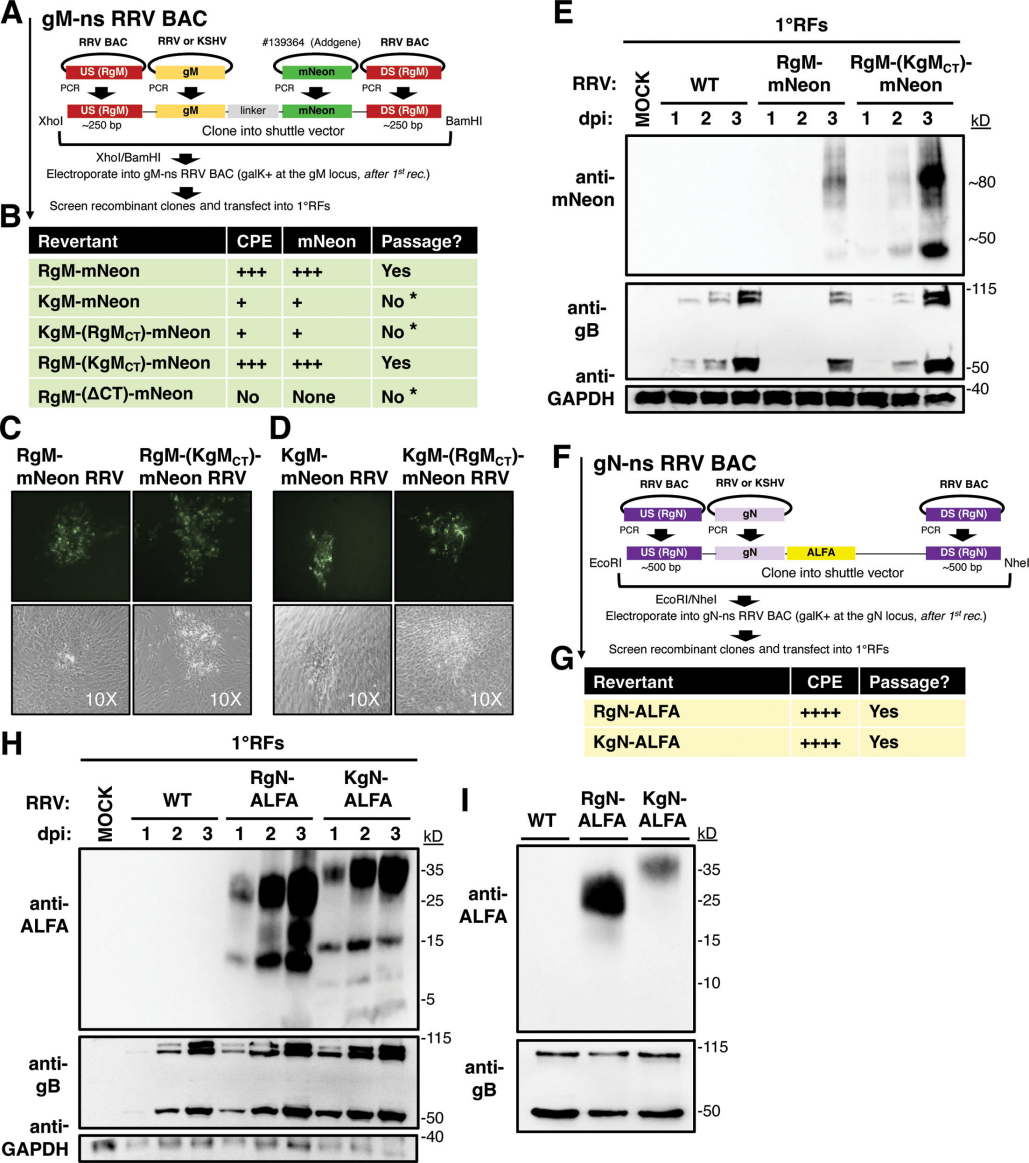
Reversion from gM-ns or gN-ns RRV BACs generated replication-competent viruses with unique properties. (**A**) Schematic for cloning and BAC mutagenesis to generate gM-revertant clones with gM-mNeon and variants with crossed CTs or with CT deleted. mNeonGreen, a GFP-like molecule, was fused to the C-terminus of each gM. (**B**) Summary of the results following transfection assays with revertant BAC clones into 1°RFs, scored qualitatively for the ability to form CPE, express gM-mNeon by fluorescence microscopy, and be passaged. Representative fluorescence microscopy of (C) the two gM-mNeon virus stocks that were successfully generated and (D) the other gM-mNeon viruses that resulted in mNeon+ cells following transfection but failed to scale up to produce virus stocks. (**E**) The viable gM-mNeon viruses were plaque purified, titered, and used to infect 1°RFs (MOI: 1.0). At 1–3 dpi, cell lysates were harvested and probed by western blot. (**F**) Schematic for cloning and BAC mutagenesis to construct gN-revertant clones, encoding a fusion with the ALFA tag (C-term.) (G) Summary of the results following the 1°RF transfection assay with revertant BAC clones, scored qualitatively for the capability to form CPE and be passaged. (**H**) Infectious gN-ALFA viruses were plaque purified, titered, and used to infect 1°RFs (MOI: 1.0). At 1–3 dpi, cell lysates were analyzed by western blot. (**I**) Gradient-purified RRVs were analyzed by western blot. *Denotes that the expansion of a given virus stock was not successful.

Earlier, we showed that the gM-ns RRV BAC was unable to spread *in vitro* and could not be propagated (Fig. 2). This raised the possibility that the stop mutations we had introduced into gM may have directly caused that null phenotype, although we could not rule out if background mutations may have occurred elsewhere in the genome during BAC mutagenesis. To investigate this, we created revertants, using the gM-ns RRV BAC as the starting material (Fig. 5A). In a previous work, using a closely related gammaherpesvirus, gM was able to support a C-terminal fusion with GFP, which did not interfere with viral growth kinetics (39), and was useful for virus binding and neutralization studies (40). Hence, we decided to try to replicate that approach, by reverting the gM-ns RRV BAC and simultaneously incorporating a GFP-like fusion at the C-terminus of gM. We decided to use mNeonGreen (over GFP) for its enhanced imaging capabilities (41). Additionally, because we knew that KgM could function in place of RgM in RRV, although somehow caused some viral attenuation, we also looked for domain-specific contributions within gM. The substantial aa differences in the gM-CTs (Fig. 1A) led us to wonder if the subpar virus growth was associated with the KgM-chi. RRV might be related to the CT. We hypothesized that KgM backcrossed with the RgM-CT (in a tail-crossed variant) might enhance viral growth. As controls, we also created other gM variants, one with RgM backcrossed with the KgM-CT, and another that lacked the CT through a deletion (ΔCT, truncation beginning at aa321).

Upon constructing and verifying these gM-mNeon RRV BACs (Fig. A3), we transfected 1^o^RFs and monitored for viral CPE, analyzed the expression of gM-mNeon by fluorescence microscopy, and attempted to develop stocks of each virus (Fig. 5B). Reversion to either RgM-mNeon or KgM-mNeon RRV was sufficient to yield mNeon+ cells as was the case for both BACs with crossed CTs (Fig. 5C and D); however, we noticed obvious disparities between them. RgM-mNeon and RgM-(KgM_CT_)-mNeon RRV BAC produced CPE and gM-mNeon (Fig. 5C) that progressed quickly, whereas CPE and gM-mNeon expression were notably decreased with the KgM-mNeon RRV BAC. This was not corrected by the KgM-(RgM_CT_)-mNeon RRV BAC, which we observed also progressed poorly. Although we did find sporadic gM-mNeon signals in the KgM-mNeon and KgM-(RgM_CT_)-mNeon RRV-transfected cells (Fig. 5D), they were limited to just a few cells and spread inefficiently over prolonged periods of monitoring, along with the much weaker CPE. Although we may have been able to passage KgM-mNeon RRV in a very limited amount, as judged by the appearance of gM-mNeon arising in newly infected cells, we were unable to develop stocks of KgM-mNeon and KgM-(RgM_CT_)-mNeon RRV (on repeated attempts). These data resonated with the strong attenuation we had defined for the KgM-chi. RRV and signified that its impairment in viral growth could potentially be associated with the body of gM (aa1-321), and not with the divergent CTs. In subsequent sections, we will continue to delineate the mechanisms responsible for this attenuation.

Interestingly, despite the striking reduction in the level of conservation between the CTs, we found that the KgM-CT could substitute for the RgM-CT, and the corresponding recombinant RRV spread efficiently in culture. We were successfully able to develop viral isolates of both RgM-mNeon and RgM-(KgM_CT_)-mNeon RRV. Upon infecting 1°RFs, these viruses displayed strong expression of the RRV antigen gB and gM-mNeon (Fig. 5E), as expected, demonstrating the first protein analyses of RRV gM, which also migrated similarly to KSHV gM in western Blot (Fig. 3C and D).

On the other hand, for the RRV BACmid lacking the CT (but with the linker and mNeon still attached at the C-terminus), neither gM-mNeon nor CPE was detected upon BAC transfection (data not shown). We suspect that the gM-CT confers an important and likely necessary role for RRV, since the CT is known to be a critical regulator of gM in other herpesviruses, including for other gammaherpesviruses (42–44). In aggregate, these reversion experiments reinforced that gM is essential for RRV to spread, demonstrated that KgM can replace RgM to support viral spread, and suggested that the gM-CT was not the structural element to explain the attenuation of KgM-chi. RRV.

### gN is essential for RRV

To determine whether the null phenotype of gN-ns RRV BAC (Fig. 2) was explicitly caused by the stop mutations introduced, and not due to background mutations, we created gN revertant RRV BACs similarly to how we had constructed the gM-mNeon RRV BACs. Starting from the gN-ns RRV BAC, we initially created gN revertants to RgN or KgN fused to an RFP-like reporter. However, this approach was not successful, perhaps because the gNs are relatively small and not able to accommodate a large fluorescent reporter. Hence, we constructed gN revertant RRV BACs fused to the ALFA tag, an ultra-high-affinity epitope tag (45). This tag was chosen because of its small size (13 aa) and its versatility for a wide range of applications.

After constructing (Fig. 5F) and validating (Fig. A4) the RgN- or KgN-ALFA RRV BACs, we again transfected 1^o^RFs, trying to reconstitute the virus. Here, we found that both ALFA-tagged RRV BACs produced viral CPE and could be passaged easily (Fig. 5G). Qualitatively, plaques associated with the RgN- and KgN-ALFA RRVs looked indistinguishable from those with WT RRV BAC. We were able to isolate each of these recombinant RRVs, which were then expanded, titered, and used to infect 1^o^RFs to analyze gN protein expression using the ALFA tag. Indeed, both ALFA-tagged RRVs expressed gN-ALFA (Fig. 5H). These revertants confirmed that gN is required for RRV spread and represent the first protein analyses of RRV gN.

Remarkably, the ALFA tag revealed at least three products for each gN fusion. Notably, the migration of each band detected by the ALFA tag was higher than expected (KgN: 11.5 kD, RgN: 11.0 kD, and ALFA: 1.8 kD). Moreover, the KSHV gN Abs did not recognize these higher molecular weight forms, only the lower molecular weight form (which was similar in size to the band detected in Fig. 3C and D). Since the epitope used to generate the KSHV gN Abs overlapped with a region of KgN that is predicted to be heavily O-glycosylated, we think that these higher molecular weight forms may be post-translationally modified, perhaps by O-glycosylation, which could interfere with Ab binding. This may explain the lack of detection with the upper molecular weight forms of gN with purified KSHV, as well as the KgN-chimeric RRVs, using the KSHV gN Abs (Fig. 3D). Although we have yet to confirm our suspicion, we found that the predominant form of gN incorporated into both RRV and KSHV are these upper molecular weight products (~25–35 kD) (Fig. 5I; Fig. 7D).

Although we cannot rule out the possibility that the KSHV gN Abs failed to recognize the higher molecular bands through a conformation-specific effect, such as being blocked by gM or its own 3-dimensional structure, we think this is less likely since we used conditions that should minimize these issues. In HCMV and the gammaherpesvirus Epstein-Barr virus (EBV), gN acquires several post-translational modifications on its ectodomain during its maturation, including O-glycosylations (46). These O-glycosylations can contribute to a significant fraction of the apparent molecular mass of gN by SDS-PAGE (26) (more than 3-fold in HCMV). If gN is indeed post-translationally modified in KSHV or RRV, not only could this explain why the KSHVgN Abs failed to efficiently detect the upper molecular weight forms of gN but could also implicate them in immune evasion by potentially shielding virions from neutralization (47).

### Glycosylation analyses of RgM

**Fig 6:**
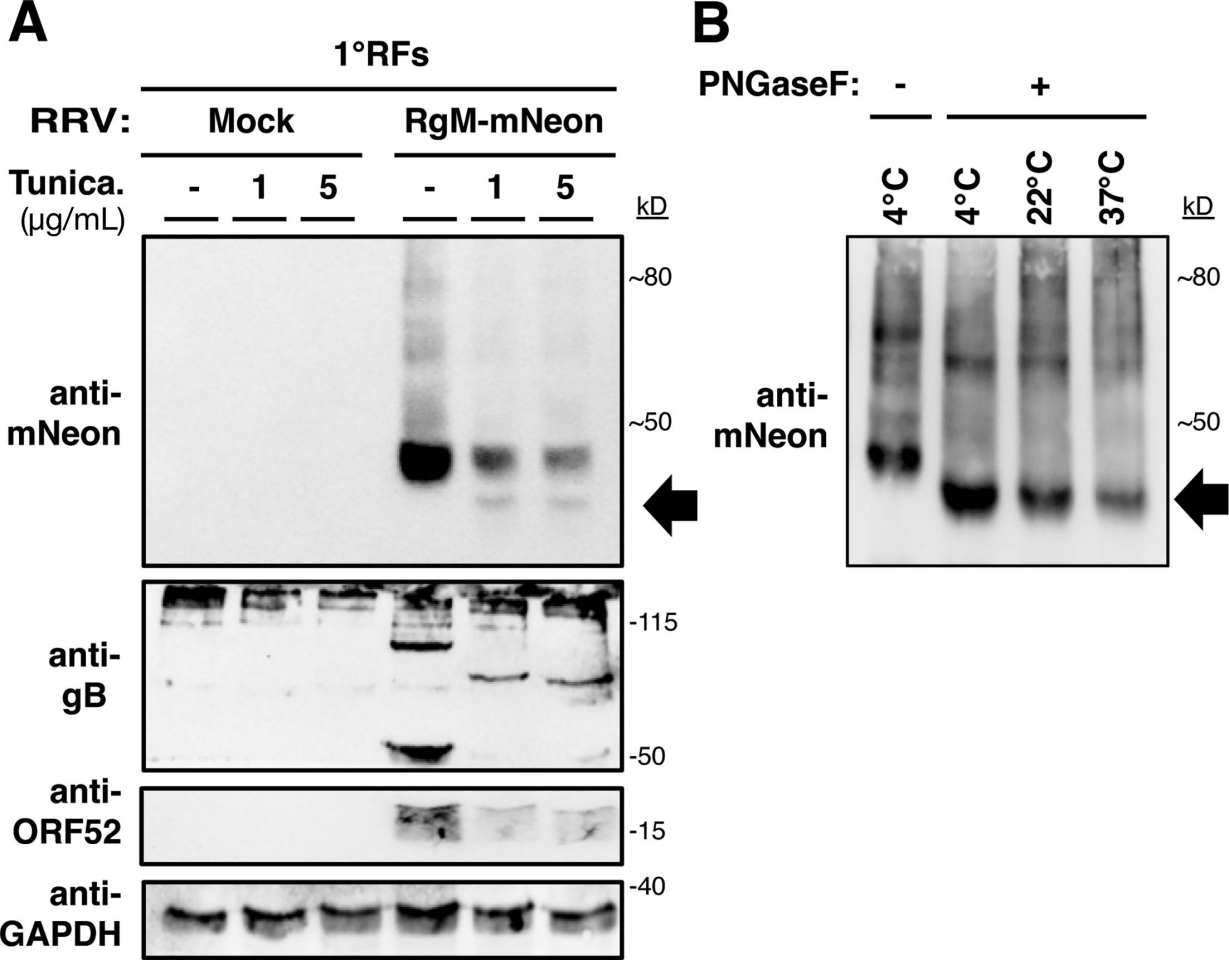
RgM is N-glycosylated. (**A**) RgM-mNeon RRV-infected 1°RFs were treated with or without tunicamycin, an inhibitor of N-linked glycosylation, and western blot was utilized to assess the migration of RgM. (**B**) RgM-mNeon RRV-infected 1°RF lysates were treated with PNGaseF to cleave N-linked glycosylations *in vitro*. The black arrow points to observed mobility shifts.

Since KgM-chi. RRV was strongly attenuated, and we knew that its phenotype was most likely associated with the body, and not the CT (Fig. 5), we wondered if this could reflect a difference in the status of N-linked glycosylation between KgM and RgM. Previously, KgM has been shown to be N-glycosylated (28), but whether RgM is also N-glycosylated was unknown. Although we identified two canonical N-linked glycosylation sites in the body of gM that are positionally conserved and identical in KgM and RgM (Fig. 1A), there are also other amino acid differences that might influence their 3-dimensional structure, which may affect the exposure and recognition of these motifs to cellular glycosylation machinery. To experimentally test if RgM is N-glycosylated, we first treated RRV-infected cells with tunicamycin, inhibiting N-linked glycosylation in cells. To supply an epitope for immunoblotting, we used RgM-mNeon RRV. We found that tunicamycin downshifted the mobility of RgM-mNeon in infected cells (Fig. 6A), as well as that of RRV gB, an internal control (38). To confirm this experiment, we then treated RgM-mNeon RRV-infected cell lysates with PNGaseF, an enzyme that cleaves N-glycosylations. Here, we also observed a mobility shift by western blot (Fig. 6B). Therefore, we concluded that like KgM, RgM can also be N-glycosylated; thus, the lack of N-linked glycosylations on RgM cannot explain the attenuation phenotype of the KgM-chi. RRV.

### RRV-chimeric KSHVs also display some impairments in viral assembly

**Fig 7:**
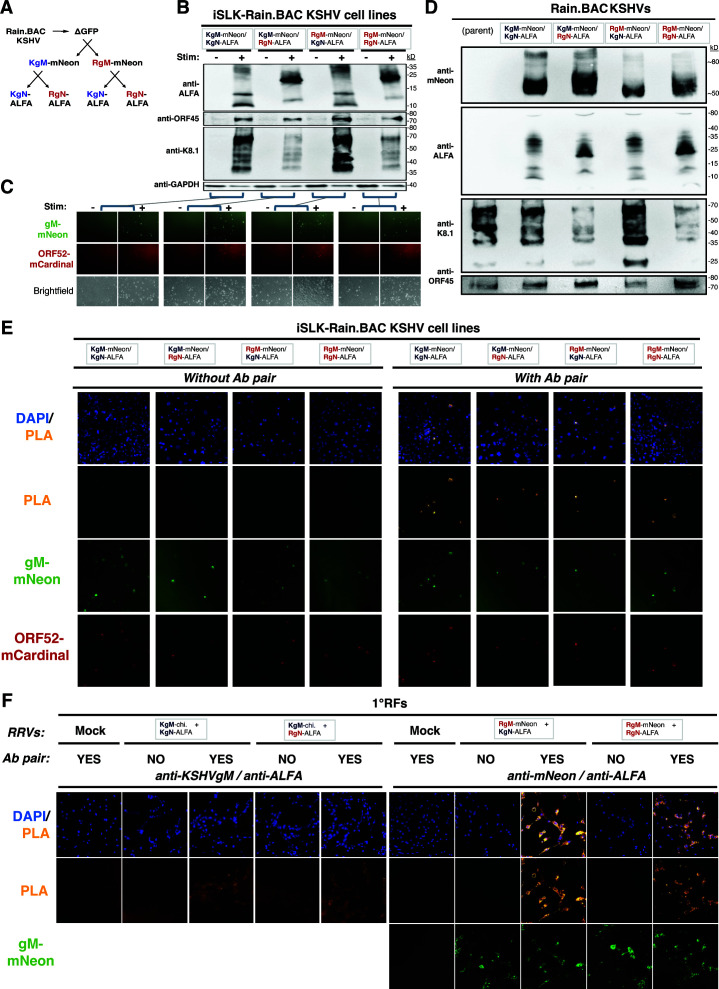
Expression and incorporation of K8.1 is diminished in RgN-chi. KSHV, despite gM/gN association. (**A**) Experimental approach to generate recombinant Rain.BAC KSHV crosses with gM-mNeon and gN-ALFA. (**B-C**) Cell lines harboring mutant Rain.BAC KSHVs were lytically reactivated and analyzed by western blot (**B**) and fluorescence microscopy (**C**). **(D**) Mutant Rain.BAC KSHVs were produced and concentrated from the cell lines and analyzed by western blot. (**E**) PLA in the KSHV cell lines using mNeon and ALFA Abs testing the formation of the gM/gN complex. (**F**) PLA in 1°RFs co-infected by RRVs using mNeon and ALFA Abs (or KSHVgM and ALFA Abs) testing for the formation of the gM/gN complex.

To determine if the attenuation of the KgM-chi. RRV was due to a gM/gN mismatch or a virus-specific defect, we generated reverse-chimeric KSHVs, by substituting RgM for KgM, RgN for KgN, or both in a KSHV BACmid. For this, we utilized the recently developed, Rainbow BAC (Rain.BAC) KSHV (48), which encodes several fluorescent markers that can be used to differentiate infected cells as they progress through the lytic cascade. Because we tagged gMs with mNeonGreen, as we had done for the RRVs, we first eliminated GFP (encoded by the parental BAC16 viral genome) by deleting it, then inserted KgM-mNeon or RgM-mNeon in place of KgM, and then used these BACs to insert KgN-ALFA or RgN-ALFA in place of KgN (Fig. 7A). After validating the BAC mutagenesis by PCR/sequencing and restriction digests (Fig. A5 and A6), we used these gM/gN crosses to establish four unique iSLK cell lines harboring the recombinant KSHVs encoding gM-mNeon and gN-ALFA from either KSHV or RRV (KgM-mNeon/KgN-ALFA [Kaleido.BAC KSHV], KgM-mNeon/RgN-ALFA [Kryptonite.BAC KSHV], RgM-mNeon/KgN-ALFA [Rocket.BAC KSHV], and RgM-mNeon/RgN-ALFA [Revolver.BAC KSHV]).

Upon lytic reactivation, all four cell lines carrying the recombinant KSHVs were capable of lytic activation and induced the expression of viral markers of reactivation, such gN-AFLA, ORF45, and K8.1 (Fig. 7B). Additionally, all four cell lines also demonstrated successful induction of gM-mNeon and the Rain.BAC-encoded marker ORF52-mCardinal by fluorescence microscopy (Fig. 7C). Although the frequency of cells expressing gM-mNeon and ORF52-mCardinal, as well as the levels of ORF45, seemed comparable, there was a striking decrease in the expression of K8.1, a major envelope glycoprotein, by the KSHVs encoding RgN. These alterations in K8.1 levels were not fully corrected by the double-chimeric KSHV, expressing both RgM and RgN, and were also present in viral stocks of RgN-chimeric KSHVs, with prominent reductions in the successful incorporation of K8.1 (Fig. 7D). This phenomenon seemed unrelated to the levels of gM or gN, as the KSHVs incorporated gM-mNeon and gN-ALFA, from either KSHV or RRV, comparably. Thus, although the mechanisms leading to this inefficient assembly of KSHV are not yet clear, RgN was less efficient than KgN in supporting K8.1 expression and incorporation into virions.

These data then raised the possibility that the KgM/RgN heterodimer, which had attenuated RRV spread and now impacted K8.1 expression in chimeric KSHV, may be unified by the inability to form a complex. To test whether the gM/gN complex could form in KSHV-infected cells, as well as for the other gM/gN crosses, we used a proximity ligation assay (PLA). Upon lytic reactivation, we found that all four KSHV cell lines were PLA-positive for the gM/gN complex and were PLA-negative in the negative controls lacking the antibodies (anti-mNeon/anti-ALFA) (Fig. 7E). As added support for these findings, the PLA signals overlapped with the gM-mNeon signals, as would be expected. To further test the gM/gN complex formation in RRV-infected cells, we performed the PLA in cells that were co-infected by different recombinant RRVs (Fig. 7F). Again, all four gM/gN pairings from KSHV and RRV were PLA-positive. Importantly, we also demonstrated that all Abs used in the PLAs recognized their intended target in cells using immunofluorescence (Fig. A7). One point to note is that despite being PLA-positive, we did observe much lower PLA signals from cells that were co-infected with KgM-chimeric RRV compared with RgM-mNeon RRV (Fig. 7F). It is difficult to determine whether these lower signals came from a reduction in gM expression with KgM compared with RgM, as we could not use mNeon to directly compare the levels of gM (since we were unable to generate stocks of the KgM-mNeon RRV). Another possible explanation is that the polyclonal KSHV gM rabbit antisera, although affinity-purified, probably has a much lower affinity compared with the monoclonal mNeon antibodies. Regardless, after considering these data, we conclude that gM/gN appear to form a complex, not only in KSHV and RRV but also in both KSHV/RRV crosses.

In sum, although the KgM/RgN pairing may be functionally defective for some aspects of viral spread in RRV and possibly viral assembly in KSHV, these consequences were independent of complex formation and could not be overcome in double-chimeric viruses, indicating that the gM/gN complexes in KSHV and RRV must have evolved in a similar, yet virus-specific manner.

## DISCUSSION

Five glycoproteins are evolutionarily conserved in all human herpesviruses—gB, gH, gL, gM, and gN. Although there is now clear evidence of the importance of gB and the gH/gL complex to many herpesviruses, the roles of gM/gN are crudely defined, especially for gammaherpesviruses.

In this report, we have demonstrated that gM and gN are essential for RRV, that gM and gN from KSHV can physically and functionally substitute for their homologs in glycoprotein-chimeric RRVs, and that there are substantial reductions in viral spread by chimeric RRVs encoding KgM, which likely mimic some defects in virus assembly by chimeric KSHVs encoding RgN. Remarkably, these alterations were not effectively surmounted by the creation of double-chimeric viruses. Thus, some aspects of these phenotypes could be virus-specific, but further studies are necessary to better define the differences between the chimeric viruses. In support of our data, we also found gM/gN heterocomplexes were able to form from KSHV, RRV, and crosses (Fig. 1C, 7E, and F)

Our findings that gM and gN were essential for RRV were not surprising. In previous work, using murine gammaherpesvirus 68 (MHV68), a related gammaherpesvirus grouped proximate to KSHV and RRV in the genus *Rhadinovirus*, gM and gN were also reported to be essential (49–51). This requirement for gM in MHV68 was first reported using random transposon mutagenesis (50). In that study, three mutants with insertions in gM were completely devoid of viral spread, and a fourth mutation in gM, located in the CT, resulted in strong attenuation of virus spread. Likewise, the requirement for gN by MHV68 was also found in a genome-wide mutagenesis screen (51). In a study focusing more specifically on gM, when stop codons were inserted into gM of MHV68 BAC and transfected into fibroblasts, the virus could not be recovered. However, a variety of revertants successfully restored virus propagation (49). Thus, in light of our data, it is now highly probable that gM and gN will be essential factors for KSHV spread as well.

In other primate gammaherpesviruses, such as EBV, gM/gN is also critical. Using insertional mutagenesis, a recombinant EBV with the gN locus disrupted was found to have severe impairments to both infectious virus production and primary infection (52). By electron microscopy, most of these viruses remained trapped in chromatin, and the majority that were released into the extracellular milieu lacked an envelope, pointing to significant defects in assembly and egress. Interestingly, in this case, although gN had been targeted, the expression of gM was also strongly compromised, indicating that there are probably many areas where gM and gN are critically co-dependent on each other. In support of this, in EBV, the co-expression of gM supports the maturation and glycoprotein processing of gN (25). Conversely, in KSHV, there is also some evidence that gN supports the maturation and post-translational modifications of gM (28). Together, these publications support the concept that gM and gN play vital and interdependent roles in gammaherpesviruses.

Since gM and gN are evolutionarily conserved in alpha- and beta-herpesviruses, additional studies could be relevant to highlight their important contributions. Similar to the consequences of removing gM or gN in RRV, these glycoproteins are also essential for the betaherpesviruses HCMV (27, 53–55) and human herpesvirus 6 (56). However, for most alphaherpesviruses, gM and gN have been found to be nonessential, with at least one exception in Marek’s disease virus, where the gM and gN homologs are both required for viral cell-to-cell spread (57). However, in several alphaherpesviruses, gM and gN can still contribute to major phenotypes in virus production and spread when they are ablated (58). For herpes simplex virus type 1 (HSV-1), when gM was deleted, plaque size was decreased considerably, and virus production, as measured by growth curves, was reduced and delayed (59). In additon, when a second deletion was introduced into UL11, a viral tegument protein, these defects were additive, indicating that the gM/gN complex may be closely integrated with viral tegument proteins, or with other viral or cellular factors in its network.

Structurally, the CTs of gM and gN have garnered particular interest as pivotal elements in unraveling the functions of these glycoproteins. In HSV-1, besides affecting plaque size, the CT of gM contributed to the distribution and envelopment of particles in the cytoplasm (60). In HCMV, the CTs of gM and gN contributed to strikingly similar, yet more consequential, phenotypes in viral assembly and secondary envelopment, whereby without the CT on gM or gN, virion morphogenesis was dramatically delayed or halted (42, 61). A multitude of defects were identified for the virus, which could not have been obtained with transient transfection assays alone. Additionally, the contribution of the CT of gM to MHV68 has also been brought to light. Although much of the CT was found to be dispensable for MHV68 replication, the most proximal part of the CT was required for the production of infectious progeny (43). Remarkably, in EBV, the gM-CT is not necessary to form a complex with gN (44). Analogously, the CTs of gM and gN do not seem to dictate gM/gN complex formation in HCMV or HSV-1 either (27, 42, 61, 62). Collectively, these data support the concept that although the gM-CT can have essential roles, possibly to help stabilize the protein (42), it is likely independent of the gM/gN complex formation.

These data are highly relevant to our work, since we were unable to recover infectious virus in a recombinant RRV lacking the gM-CT and considering the outcomes with the gM-CT crosses. Due to the extremely high amino acid divergence between KgM-CT and RgM-CT, we hypothesized that we could swap the RgM-CT back onto the KgM-chi. RRV to reverse the attenuation phenotype. Not only did this not work, but to our astonishment, we successfully recovered the inverse virus, an RRV encoding RgM with the KgM-CT. These findings may provide some evidence that despite the divergence in protein sequence, the KgM and RgM CTs might have some degree of structural or functional homology, which may be useful as a starting place for future studies to advance our broader understanding of gM-CTs.

In terms of analyzing the chimeric RRVs compared with chimeric KSHVs, it is important to acknowledge the limitation that we reached our conclusions using very different models. The RRV model is based on primary infection, whereas the KSHV model reported here is based on lytic reactivation under artificial conditions. It would certainly be interesting to see if these findings could be replicated using primary infection models that have recently been developed for KSHV (63).

Nevertheless, our data strongly implicate that the gM/gN complex is a key regulator of virus production and spread. Our data precipitated the possibility that the KgM-chi. RRV might have been attenuated due to an inability to form the KgM/RgN complex. However, we found that KgM/RgN, and the other gM/gN combinations, are capable of binding; hence, it is highly likely that the KgM/RgN pairing affects RRV and KSHV in other ways. Future work should be carried out to dissect whether and how gM/gN bridges with key tegument proteins during viral assembly. A better understanding of these virus protein interactions may reveal a deeper appreciation of the key drivers of infectious virus production and spread, probably at later stages of infection and during viral envelopment, which could also support innovative advances in vaccine development.

Currently, KSHV vaccine candidates have not yet reached clinical studies; however, in recent years, these efforts have been gaining momentum, fostered by the development of new vaccine technologies and platforms, as well as by innovative vaccine approaches. One vaccine strategy that could be considered is a replication-defective KSHV, which has been modeled elegantly using MHV68 (64, 65). Although promising, a virus-inactivation strategy might face potential safety concerns if translated to KSHV. Another vaccine strategy could be to use a viral vector, incorporating one or more KSHV antigens of interest. When such experimental vaccines have been administered to mice or rabbits, neutralizing antibodies were elicited that could block KSHV infection *in vitro* (66, 67). Moreover, nonviral nanoparticle platforms have also been examined (68). However, ultimately, whether these successes can translate into effective options to protect humans is unclear.

Taken together, with these KSHV and RRV glycoprotein-chimeric viruses, gM and gN can now be evaluated for the first time as vaccine targets for KSHV, both *in vitro* and *in vivo*. If applied to NHP animal models, these viruses may not only deepen our understanding of these critical proteins during viral infection but may also guide the development of experimental vaccines, alongside other vaccine targets, from preclinical testing to clinical trials.

## MATERIALS AND METHODS

### Cells

Primary rhesus fibroblasts (1^o^RF) and iSLK cells were grown in Dulbecco’s modification of Eagle’s medium (DMEM) (Corning, Manassas, VA) supplemented with 10% fetal bovine serum (FBS) (HyClone, Logan, UT), 100 U/mL penicillin, and 100 µg/mL streptomycin. Brk219 (BJAB-KSHV) cells (69) were grown in RPMI and supplemented similarly. The iSLK cells that support KSHV reactivation have been described previously (70) and were maintained in 1 µg/mL puromycin and 250 µg/mL G418.

### Computational analyses

Alignments were performed by NCBI BLASTp. Membrane topology predictions were performed by DeepTMHMM (https://dtu.biolib.com/DeepTMHMM). Glycosylation predictions were made by NetNGlyc-1.0 (https://services.healthtech.dtu.dk/services/NetNGlyc-1.0) and NetOGlyc-4.0 (https://services.healthtech.dtu.dk/services/NetOGlyc-4.0). gM/gN protein complexes were modeled by AlphaFold Colab, a neural network-based prediction tool that runs a simplified version of AlphaFold (v2.3.2) that can also generate structural predictions of dimers (36, 37). The gN signal peptides (aa1-23) were predicted using SignalP 5.0 (https://services.healthtech.dtu.dk/services/SignalP-5.0) and omitted from structural predictions of the gM/gN complexes. Physical representations of the protein complexes were rendered using PDB-3D viewer.

### Construction of recombinant RRVs

Recombinant RRVs were constructed using RRV 17577, an infectious and pathogenic strain of RRV isolated from a RM with a lymphoproliferative disorder (12), which was subsequently cloned as a BACmid (71).

All recombinant RRVs were constructed using *galK* homologous recombination in SW105 *Escherichia coli* (72), similar to what has been reported before (73). In short, recombinant clones were first positively selected by their ability to grow on minimal media, containing galactose, conferred by electroporation and insertion of a *galK* cassette into the RRV genome at a targeted locus (either gM or gN), amplified from the plasmid pGALK, using primers containing 40 bp of homology that flank each side of the targeted locus. To construct final recombinant clones, shuttle plasmids were developed that included 250–500 bp of the flanking region of RRV, plus the KSHV gene or nonsense mutation to be inserted. These shuttle plasmids were created by overlap-extension PCR and standard cloning methods. The repair DNA fragment was excised out of the shuttle plasmids and electroporated into the galK^+^ clones. The removal of the *galK* cassette was then selected in the presence of 2-deoxygalactose. Successful RRV recombinants were confirmed by PCR/sequencing and restriction digest/southern blot.

For southern blot analyses, BACmid DNA was digested overnight with NdeI, then electrophoresed slowly (~23 V) into a 0.8% agarose gel. The digested DNA was then transferred to nitrocellulose membranes by vacuum transfer, and the membranes were probed by hybridization with digoxigenin (DIG)-labeled PCR products specific to various viral genomic regions of interest. DIG labeling was performed following the protocol from the manufacturer (DIG-High Prime kit, cat. 11745832910 MilliporeSigma, Burlington, MA).

### Generation of recombinant RRVs

To produce the initial stocks of recombinant RRVs, recombinant RRV BAC DNA was transfected into 1^o^RFs, seeded the day prior, using TransIT-LT1 (Mirus Bio, Madison, WI) and freshly prepared BAC DNA (isolated from a 5 mL overnight culture and resuspended in 40 µL of water). We transfected 10 µL, or about 1 µg, of BAC DNA per well of a six-well plate. Signs of viral CPE were then monitored regularly using a light microscope. To maximize virus yield, at ~1 month post-transfection (or earlier with strong CPE), the cells were scraped and sonicated twice (30 s each cycle, at medium power output), and the supernatants were pooled with the original supernatants, clarified by a low-speed spin to remove cells and cell debris, and these inocula were then used to infect new 1^o^RFs for subsequent rounds of viral passaging and expansion.

In our founding experiments, to better characterize gM- and gN-nonsense RRV mutants, we also performed DNA, RNA, and protein analyses of BAC-transfected cells at weekly intervals. After 2 days post-BAC transfection, the media were removed, and the cells were washed twice in PBS and then supplied with fresh media. For DNA, the cells were lysed in nuclei lysis buffer (Promega, Madison, WI). For RNA, the cells were lysed in RNA lysis buffer, as part of the Quick-RNA miniprep kit (Zymo Research, Irvine, CA), and for proteins, the cells were lysed in RIPA buffer (50 mM Tris-HCl, 150 mM NaCl, 0.1% SDS, 0.5% sodium deoxycholate, 1.0% IGEPAL-630), complemented by 1× protease inhibitor cocktail (MilliporeSigma, cat. P8340).

### Isolation of recombinant RRVs

For all recombinant RRVs described in this paper, after obtaining the infectious virus, and before proceeding to additional experiments, the BAC cassette (flanked by LoxP sites in the RRV BAC genome) was first excised by CRE/Lox recombination. Briefly, 1^o^RFs were transfected with a CRE expression plasmid and then infected by the working stock of recombinant RRV. This step was performed at least twice, then the working stock of recombinant virus was plaque purified two times, and the successful removal of the BAC cassette was verified by PCR/sequencing. Verification sequencing of the final viral isolates was also performed by PCR and conventional sequencing. To analyze the viral genome for KgM-chi., KgN-chi., and KgMgN-chi. RRVs, we also performed sequencing by Illumina.

### Purification and titration of RRV

RRV stocks were purified from 2 to 3 RRV-infected roller bottles of 1°RFs. In the first phase of purification, virus supernatants were separated from the cell fraction by a short, low-speed centrifugation, followed by a high-speed centrifugation to concentrate the virus (12,000 × *g* for 1 h). In the second phase of purification, the cell-associated fraction was freeze/thawed and sonicated twice (30 s each cycle, at medium power output) to release intracellular virus. Clarified supernatants were then pooled with the other virus fractions, and the virus was ultracentrifuged through a 30% sorbitol cushion using a Beckman SW41Ti rotor (40,000 × *g* for 1 h), and then resuspended in 2 mL of DMEM, aliquoted, and frozen at −80°C.

To titer RRV, we performed plaque assay, similar to what has been described previously (74). Cells were seeded into six-well plates (2 × 10^5 cells per well). On the next day, they were infected with serial dilutions of the virus for 2–3 h (in 0.5 mL) at 37°C, slowing rocking. Afterward, a methylcellulose overlay was added to the cells (containing 1× DMEM, 10% FBS, 100 U/mL penicillin, 100 µg/mL streptomycin, 1% methylcellulose, and 1% sodium bicarbonate). The plates were incubated for ~12 days at 37°C, before 2 mL of neutral red (0.01% diluted in PBS) was added to the top of the methylcellulose overlay to counterstain the cells. A day later, the number of plaques was manually counted with the aid of a light microscope.

### *In vitro* growth curves

To analyze the rate of growth of RRV, we used an assay previously described (75). For this, 1^o^RFs were seeded into cell culture tubes (Greiner Bio-One, cat. 191160) at a density of 1 × 10^5 cells per tube, on a 20° tilt. One day post-seeding, 1^o^RFs were infected at an MOI of 0.1 for 2–3 h in the 37°C incubator. After absorption, the virus was removed, and the cells were washed twice with PBS and then supplied with fresh media. At each time point post-infection, the tubes were frozen at −80 °C. Later, these tubes were thawed at room temperature, incubated on ice, and sonicated for two cycles at medium power output to release intracellular viruses. After supernatants were clarified from cells and cell debris, RRV titers were measured by plaque assay.

### Cloning gM-mNeon and gN-ALFA shuttle plasmids for epitope-tagged RRVs

To construct recombinant RRVs with C-terminally tagged gM-mNeon or gN-ALFA, shuttle plasmids were assembled using overlap-extension PCR and standard cloning methods. The gM-mNeon shuttle plasmids were cloned using Addgene plasmid #139364 as a template for mNeon, with a 12-aa linker (GSAGSAAGSGEF) amended by including a corresponding nucleotide sequence in the primers. To clone the gN-ALFA shuttle plasmids, nucleotides encoding the ALFA tag (SRLEEELRRRLTE), preceded by one proline spacer, were included in the cloning primers.

### Construction of recombinant KSHVs

To construct recombinant KSHV, *en passant* BACmid mutagenesis was performed using GS1783 competent cells, which has been described previously (76, 77). For our experiments, we used the Rainbow BAC KSHV (48). However, we first deleted the GFP open reading frame, which is normally constitutively expressed from the KSHV BAC genome, under the control of an EF1⍺ promoter, as this signal would interfere with the fluorescence associated with gM-mNeon-tagged KSHVs. Then, with the ΔGFP-Rainbow BAC KSHV as the starting material, we inserted the mNeon fusion (preceded by the same 12-aa linker used for the recombinant RRVs) at the C-terminus of gM (for KgM-mNeon KSHV) or replaced the KSHVgM open reading frame with RRVgM-linker-mNeon (for RgM-mNeon KSHV). Kanamycin shuttle plasmids (KgM-Kan-mNeon and RgM-Kan-mNeon) were assembled using plasmid pEP-KanS as a template. These plasmids served as a template for PCR, from which linear PCR products were DpnI-treated and used to electroporate GS1783-carrying KSHV BACs for mutagenesis. Insertion and resolution of the Kan cassette in recombinant KSHV BAC clones were validated by PCR/sequencing and ScaI restriction digestion analyses. Similar methods were employed to create gN-ALFA-tagged KSHV BACs.

### Generation and reactivation of KSHV cell lines

Recombinant Rain.BAC KSHV were transfected into iSLK cells using TransIT-LT1. Two to 3 days post-BACmid transfection, the cells were trypsinized and reseeded in 0.3 mg/mL hygromycin (along with 1 µg/mL puromycin and 250 µg/mL G418) to begin selection for KSHV-infected cells. About 1 week later, the cells were split again, and hygromycin was increased to 0.5–0.6 mg/mL. At the following split, hygromycin was increased to its full maintenance concentration of 1 mg/mL. Cell lines were considered complete after ~1 month in hygromycin.

To test for lytic reactivation, iSLK cells harboring recombinant KSHV were seeded into six-well plates, and on the next day, the media was changed to 1 µg/mL doxycycline (Dox) and 1 mM sodium butyrate (NaB). Reactivation for gM-mNeon and mCardinal-ORF52 in Rainbow BAC KSHV was monitored in live cells by fluorescence microscopy (dpi 3) and in cell lysates by western blot (dpi 5).

### Purification of KSHV

To produce a large stock of KSHV, for each virus, we seeded 10, T150s with the iSLK cell lines harboring recombinant Rain.BAC KSHV. On the next day, these cells were triggered into lytic reactivation using 1 µg/mL Dox and 1 mM NaB. Cells were incubated at 37°C for ~96 h, then the supernatants were collected and cleared of cell debris by low-speed centrifugation. Additionally, the cells were sonicated (two cycles at medium power output) to release intracellular virus, and all clarified supernatants were pooled and vacuum-filtered through a 0.45 μm PES membrane. The virus was initially concentrated by spinning at 12,000 × *g* for 2 h (4°C), then purified through a 30% sorbitol cushion using a Beckman SW32Ti rotor at 90,000 × *g* for 2 h (4°C), resuspended in 0.5 mL of DMEM (concentrating the virus ~400-fold compared with the original volume), and aliquoted and frozen at −80°C.

### Proximity ligation assay

To visualize the PLA signals, as well as gM-mNeon localization at higher magnification, KSHV- or RRV-infected cells were fixed in 4% paraformaldehyde, washed twice with PBS, and then permeabilized with 0.4% Triton X-100. The PLA was performed using cells seeded and reactivated (for KSHV) or co-infected (for RRV) in an 18-well µ-slide chamber (ibidi, Munich, Germany) according to the protocol from the manufacturer (Duolink *In Situ* Orange Kit for Mouse/Rabbit, cat. DUO92102, MilliporeSigma) and imaged by confocal microscopy. For KSHV-infected cells, PLA was assessed in iSLK-KSHV cell lines 3 days post-reactivation (1 µg/mL Dox and 1 mM NaB). For RRV-infected cells, PLA was assessed in 1°RFs 1 day after being co-infected by two RRVs (each at an MOI of 1.0).

### Antibodies and chemicals

The anti-RRV Abs, anti-ORF52 (clone 3G9.2), anti-MCP (clone 3D1.2), and anti-gB (clone 1E1.2) were all generated previously from hybridomas, the fusion products of plasma cells from mice immunized with gradient-purified RRV virions. The recognition of these Abs for specific RRV targets has been internally validated previously. Anti-K8.1 (clone 4A4) and anti-GAPDH (sc-51906) Abs were obtained from Santa Cruz Biotechnology (Dallas, TX). Anti-mNeonGreen (clone 32F6) was purchased from Proteintech (Rosemont, IL). Anti-ALFA, a camelid single-domain recombinant protein fused to either mouse IgG1 Fc (N1582) or rabbit Fc (N1583), was acquired from NanoTag Biotechnologies (Göttingen, Germany). Tunicamycin (cat. 50–202-9378) was supplied via Medchemexpress (Monmouth Junction, NJ). PNGaseF (cat. P0709S) was purchased from New England Biolabs (Ipswich, MA) and used as recommended by the manufacturer, with the only exception being that RRV-infected cell lysates were incubated overnight with PNGaseF at different temperatures, as too much heat may cause gM to aggregate and/or degrade. Dox and NaB were purchased from MilliporeSigma (cat. D9891 and B5887). Anti-human IgM (μ-chain specific), used for the reactivation of Brk219 (BJAB-KSHV) cells, was obtained from MilliporeSigma (cat. I0759).

### Development of KSHV gM and gN antibodies

The anti-KSHVgM and anti-KSHVgN Abs were sourced through Pacific Immunology (Ramona, CA). There, New Zealand white rabbits were immunized with a Cys-tagged gM peptide (PAPRTQYQSDHESDSEIDET, aa376-395 of KgM) targeting the CT, or a Cys-tagged gN peptide (STERSSPSTAGLSARPSPGPT, aa31-51 of KgN) targeting the ectodomain. Both peptides were delivered in Complete Freund’s Adjuvant. At 3, 6, and 10 weeks post-primary immunization, the rabbits were boosted by re-immunizing with the same peptides, only resuspended in incomplete Freund’s adjuvant. The antisera were collected over the course of several bleeds, and antisera collected at 11 weeks post-primary immunization were used to affinity purify KSHVgM and KSHVgN Abs, which were then validated by ELISA and subsequently used for biochemical assays.

### Lysate preparation and western blot

To detect viral protein expression by western blot, we found that routine methods did not work well for the highly hydrophobic protein gM and were equally challenging for gN. Therefore, we transitioned away from lysing cells using the rather harsh RIPA buffer, in favor of other extraction methods that may favor better extraction/solubilization of gM and gN. Our methodology was developed based on earlier work on HCMV gM (26). To detect gM and gN, the cells were scraped off the tissue culture vessels and pelleted, washed with PBS, and then lysed in ice-cold Transmembrane Protein Extraction Reagent (TMB) (Fivephoton Biochemicals, San Diego, CA) with 1× protease inhibitor cocktail. The tubes were vortexed vigorously for 15 s, and every 15 min for an hour. Then, the lysates were centrifuged at 16,000 × *g* for 10 min (4°C) to pellet insoluble material. The soluble fraction was transferred to new Eppendorf tubes, aliquoted, and frozen, either at −80°C or −20°C.

To detect gM, we combined these precleared, TMB lysates (or virus preparation) with sample buffer containing: 15 mM Tris-Cl, 4% SDS (wt/vol), 16% glycerol (vol/vol), 0.01% bromophenol blue, 4%–8% β-mercaptoethanol (vol/vol), and 8 M urea. After combining these at a 1:1 ratio, the samples were incubated at room temperature for at least 15 min before loading into gels (as heating can cause the loss of gM due to aggregation). A special technique that we used for gM detection was to electrophorese the lysates through SDS-PAGE gels containing 3 M urea and 0.5% Triton X-100 (vol/vol) (present in both stacking and separating gels). After electrophoresis, semi-dry transfer was used to transfer gM proteins, as well as most proteins (besides gN proteins) to a PVDF membrane.

To detect gN (and other proteins), precleared, TMB lysates (or purified virus) were also combined 1:1 with the sample buffer described above (but without urea). The tubes were then vortexed vigorously, and incubated at 55°C for 10 min. (For all other proteins, the samples were simply heated at 95°C for 10 min.) For gN detection, the lysates were electrophoresed through a 10%–20% tricine gel (ThermoFisher, Waltham, MA) at 150V for ~1 h, which provided the highest resolution due to the size of the gN proteins. Additionally, to further maximize the efficiency of protein transfer, gN proteins were transferred to PVDF membranes using wet transfer.

After transfer, PVDF membranes were blocked in 5% milk, and diluted in 1× tris-buffered saline with 0.1% Tween-20 (TBST) for at least an hour at room temperature. Membranes were then incubated with primary Abs (typically 1:1,000, diluted in 10% blocking buffer) overnight at 4°C. The following day, the membranes were washed three times in TBST (each 5–10 min), incubated with HRP-conjugated secondary Abs (1:2,000 – 1:5,000, diluted in 10% blocking buffer) for 1 h at room temperature, then washed three times in TBST (each 5–10 min), and developed using the HRP-reactive chemiluminescent substrate SuperSignal West Pico PLUS (ThermoFisher).

### RT-PCR

RNA was extracted from virus-infected cells using the Quick-RNA miniprep kit (Zymo Research, Irvine, CA) and treated with DNAseI. RNA was cleaned and concentrated by spin column (Zymo Research). Total RNA was measured by a Nanodrop (ND-1000) UV-vis spectrophotometer, and 140–180 ng of RNA was used to generate a cDNA library using SuperScript III First-Strand Synthesis SuperMix (Invitrogen, Carlsbad, CA), with or without RT. Then, 1–5 μL of each cDNA library was tested for PCR amplification using the following primers (5’−3’):

KSHVgM (Fwd: CTGTACCTATCAACAACCGCC, Rev: AAAAGCCTACCTGCCGTCTC);

KSHVgN (Fwd: GTGTCGCATCATCTACAGAG, Rev: ACAAGCAACGTCATAGAATCC);

vMIP (Fwd: CCTATGGGCTCCATGAGC, Rev: ATCGTCAATCAGGCTGCG);

GAPDH (Fwd: AAGCCCCATCACCATCTTCCAG, Rev: ATGAGTCCTTCCACGATACCAAAG).

### qPCR

DNA was isolated using the PureGene DNA extraction kit (Qiagen, Valencia, CA). Total DNA was measured using a NanoDrop spectrophotometer and then diluted to 10 ng/µL. RRV viral loads were quantified using 100 ng of total DNA per well using a TaqMan 96-well PCR assay, as previously reported (71), using RRV (vMIP)-specific primers. Amplifications were carried out on a QuantStudio 3 Real-Time PCR machine (Applied Biosystems). qPCR for RRV-infected cells and supernatants was performed in biological and technical duplicates. For normalization, RRV viral loads were analyzed relative to the housekeeping gene GAPDH. To ensure that supernatants were processed equivalently during the isolation of viral DNA, after a low-speed spin (400–600 × *g* for 5 min) to remove cells, each sample was spiked with 600 ng of RRV-negative 1^o^RF DNA.

### Microscopy

To visualize gM-mNeon and ORF52-mCardinal signals in live, KSHV-, or RRV-infected cells, we utilized a Zeiss Axiovert 25 inverted light/fluorescence microscope, fitted with green and red filters and a Zeiss HBO 50 mercury bulb illuminator for fluorescence. Images were captured using a 2.0 MP Infinity2 digital camera from Lumenera (Teledyne Technologies, Thousand Oaks, CA).

To visualize PLA signals, as well as gM-mNeon at higher magnifications, KSHV- or RRV-infected cells were imaged using confocal microscopy. Images were acquired on a Leica Stellaris8 inverted microscope (Leica DMi8), controlled with Leica Application Suite X (built 4.5.0.25531), using a HC PL APO CS2 20×/0.75 Dry objective (Leica). Z stacks were captured using the system-optimized settings. The resolution for the HC PL APO CS2 20×/0.75 Dry objective with a pixel resolution of 1.137 µm (XY) and 0.685 µm (Z). DAPI was excited with a 405-diode laser with an emission range from 425 to 508 nm (Gain 12.6/Offset 0) for KSHV-infected cells and from 416 to 465 nm (Gain 26.2/Offset 0) for RRV-infected cells. The other fluorophores were excited using a White Light Laser (440–790 nm, set to 85.00% maximum power) with excitation wavelengths set to: 504 nm for mNeonGreen (emission range 509–540 nm [Gain 39.2/Offset 0]), 554 nm for Duolink PLA Orange (emission range 559–631 nm [Gain 3.1/Offset 0]), and 600 nm for mCardinal (for KSHV-infected cells only) (emission range: 658 nm–834 nm [Gain 61.2/Offset 0]).

For immunofluorescence experiments, images were also acquired on the same Leica Stellaris8 inverted microscope described above, using a HC PL APO CS2 20×/0.75 Dry objective (with a pixel resolution of 1.137 µm [XY] and 0.685 µm [Z]) or an HC PL APO CS2 40 ×/1.30 Oil objective (with a pixel resolution of 0.569 µm [XY] and 0.346 µm [Z]). DAPI was excited with a 405-diode laser with an emission range from 425 to 509 nm (Gain 7.6/Offset 0). The other fluorophores were excited using a White Light Laser (440–790 nm, set to 85.00% maximum power) with excitation wavelengths set to 504 nm for mNeonGreen (emission range 509–600 nm [Gain 35.5/Offset 0]) and 595 nm for Texas Red (emission range [600 nm–750 nm] [Gain 13–37.1/Offset 0]).

For both PLA and immunofluorescence experiments, a pinhole size of 1 AU (Emission Wavelength for PinholeAiry Calculation 580 nm) was applied (Pinhole 56.7 µm), and images were acquired using Power HyD S detectors (Leica) in sequential scanning mode (scan speed set to 400 Hz).

### Statistical analysis

Repeated measure analysis of variance, mixed model, was used to evaluate recombinant BACmid transfections over time on viral genome copies. First-order autoregressive (AR1) was chosen to be a within covariance structure using Bayesian Information Criteria (BIC). There was significant interaction between recombinants and time (*P* < 0.0001). That is recombinant differences changed over time. As seen in Fig. 2B and E, there were recombinant differences that can be seen at weeks 2 and 3, and the magnitude of the difference is getting larger with time. Since these comparisons were made with a very small number of biological replicates (*n* = 2) and technical replicates (*n* = 2), these findings should not be confirmatory. but rather should be purely considered exploratory.

## Data Availability

The following KSHV-chimeric RRV genomes have been sequenced and deposited into Genbank (NCBI) using the following accession numbers– PQ507925: KSHVgM-chimeric RRV (KgM-chi. RRV), PQ507926: KSHVgN-chimeric RRV (KgN-chi. RRV), PQ507927: KSHVgMgN-chimeric RRV (KgMgN-chi. RRV, with KgM-L10I), and PQ507928: KSHVgMgN-chimeric RRV (KgMgN-chi. RRV, with BAC cassette present).

## SUPPLEMENTAL MATERIAL

Supplemental material jvi.01922-24-s0001.pdf Figures A1 to A7.

ASM does not own the copyrights to Supplemental Material that may be linked to, or accessed through, an article. The authors have granted ASM a non-exclusive, world-wide license to publish the Supplemental Material files. Please contact the corresponding author directly for reuse.
