# Supplementary material for: Glycoproteins gM and gN are indispensable factors for rhesus macaque rhadinovirus replication and spread but can be reconstituted by KSHV chimeras
Source: J Virol. 2025 Feb 25;99(3):e01922-24. doi: 10.1128/jvi.01922-24 (PMC11915806; doi:10.1128/jvi.01922-24)
Supplement: Supplemental material — Figures A1 to A7. [file jvi.01922-24-s0001.pdf]

Appendix:

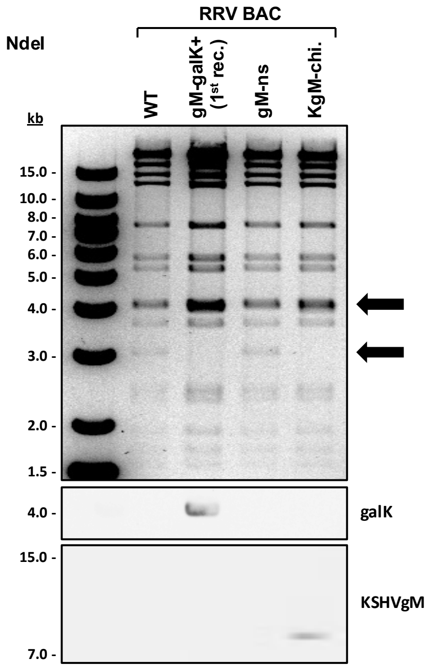

Fig.A1. Confirmation of the generation of gM-ns and KgM-chimeric RRV.
Recombinant RRV BAC DNA was digested by NdeI, analyzed by gel electrophoresis, then probed via Southern blot–first with a probe specific to galK, then the blot was stripped and hybridized with a probe specific to KSHVgM. The black arrows point to the expected mobility shifts due to the recombinations.

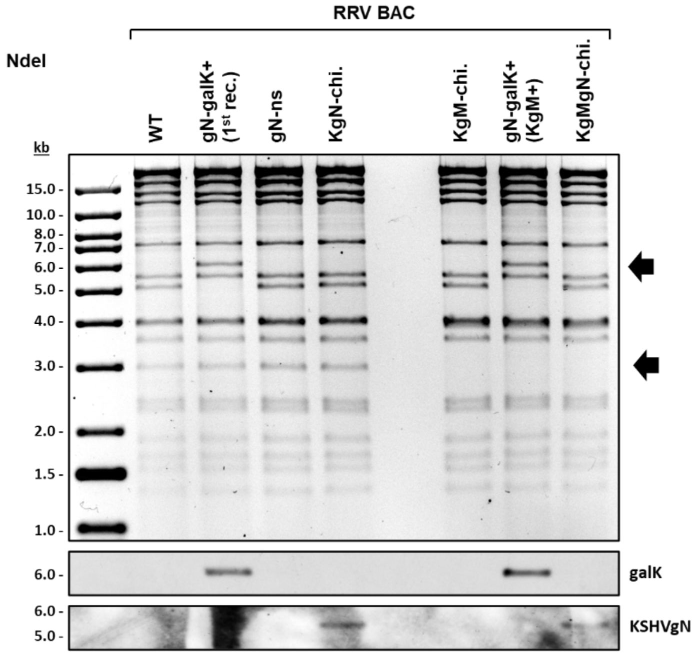

Fig.A2. Confirmation of the generation of gN-ns, KgN-chimeric RRV, and KgMgN-chimeric RRV.
Recombinant RRV BAC DNA was digested by NdeI, analyzed by gel electrophoresis, then probed via Southern blot–first with a probe specific to galK, then the blot was stripped and hybridized with a probe specific to KSHVgN. The black arrows point to the expected mobility shifts due to the recombinations.

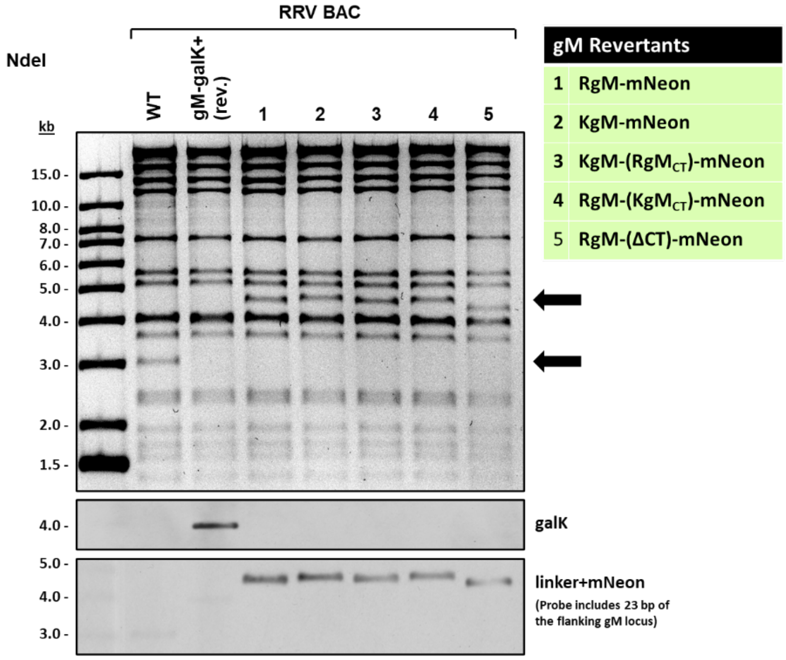

Fig.A3. Confirmation of the generation of gM revertants encoding gM-mNeon variants in RRV BAC. Recombinant RRV BAC DNA was digested by NdeI, analyzed by gel electrophoresis, then probed via Southern blot– first with the galK probe, then the blot was stripped and hybridized with an mNeon-targeted probe. The black arrows point to the expected mobility shifts due to the recombinations.

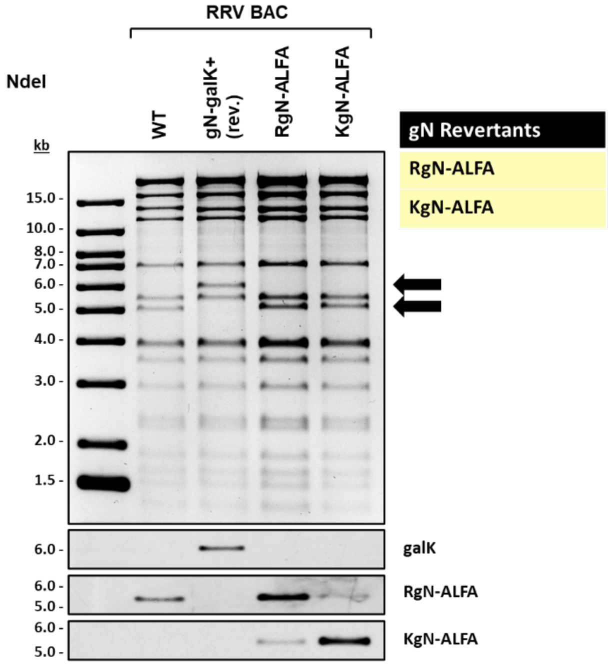

Fig.A4. Confirmation of the generation of gN revertants encoding gN-ALFA in RRV BAC. Recombinant RRV BAC DNA digested by NdeI, analyzed by gel electrophoresis, then probed via Southern blot– first with the galK probe, then the blot was stripped and hybridized with an RgN-ALFA-targeted probe, then the blot was re-stripped and hybridized with an KgN-ALFA-targeted probe. The black arrows point to the expected mobility shifts due to the recombinations.

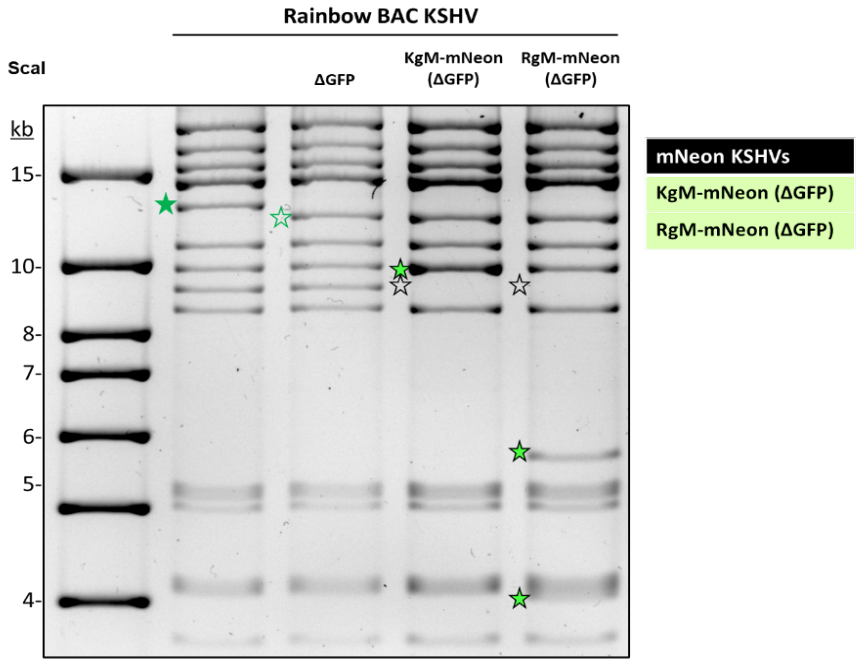

Fig.A5. Confirmation of the generation of gM-mNeon fusions encoded by Rain.BAC KSHV.
Recombinant Rain.BAC KSHV was deleted for GFP, then used to derive KgM-mNeon or RgM-mNeon Rain.BAC KSHV. Corresponding BACmid DNA was digested by ScaI and analyzed by gel electrophoresis. The stars indicate the expected mobility shifts due to the recombinations.

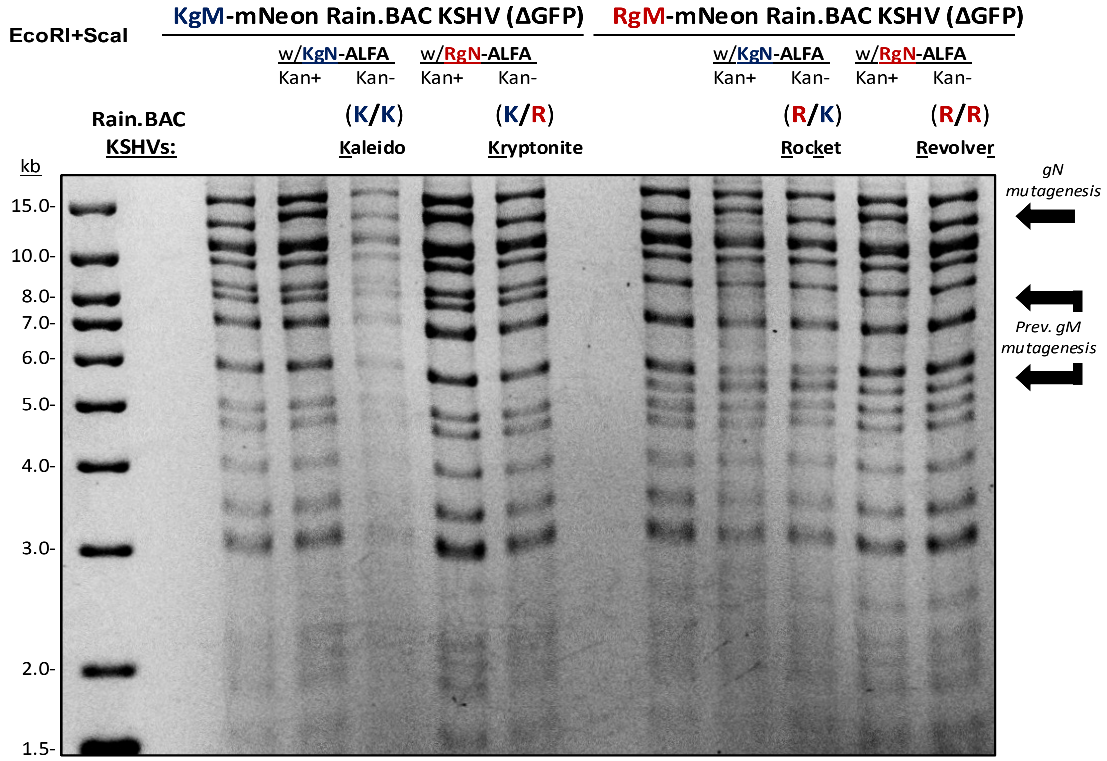

Fig.A6. Confirmation of the generation of gM/gN Crosses in Rain.BAC KSHV.
KgM-mNeon or RgM-mNeon Rain.BACs were used to derive: Kaleido.BAC KSHV (KgM-mNeon/KgN-ALFA), Kryptonite.BAC KSHV (KgM-mNeon/RgN-ALFA), Rocket.BAC KSHV (RgM-mNeon/KgN-ALFA), and Revolver.BAC KSHV (RgM-mNeon/RgN-ALFA). Corresponding BACmid DNA was double digested by ScaI/EcoRI and analyzed by gel electrophoresis. The black arrows point to the expected mobility shifts due to the recombinations.

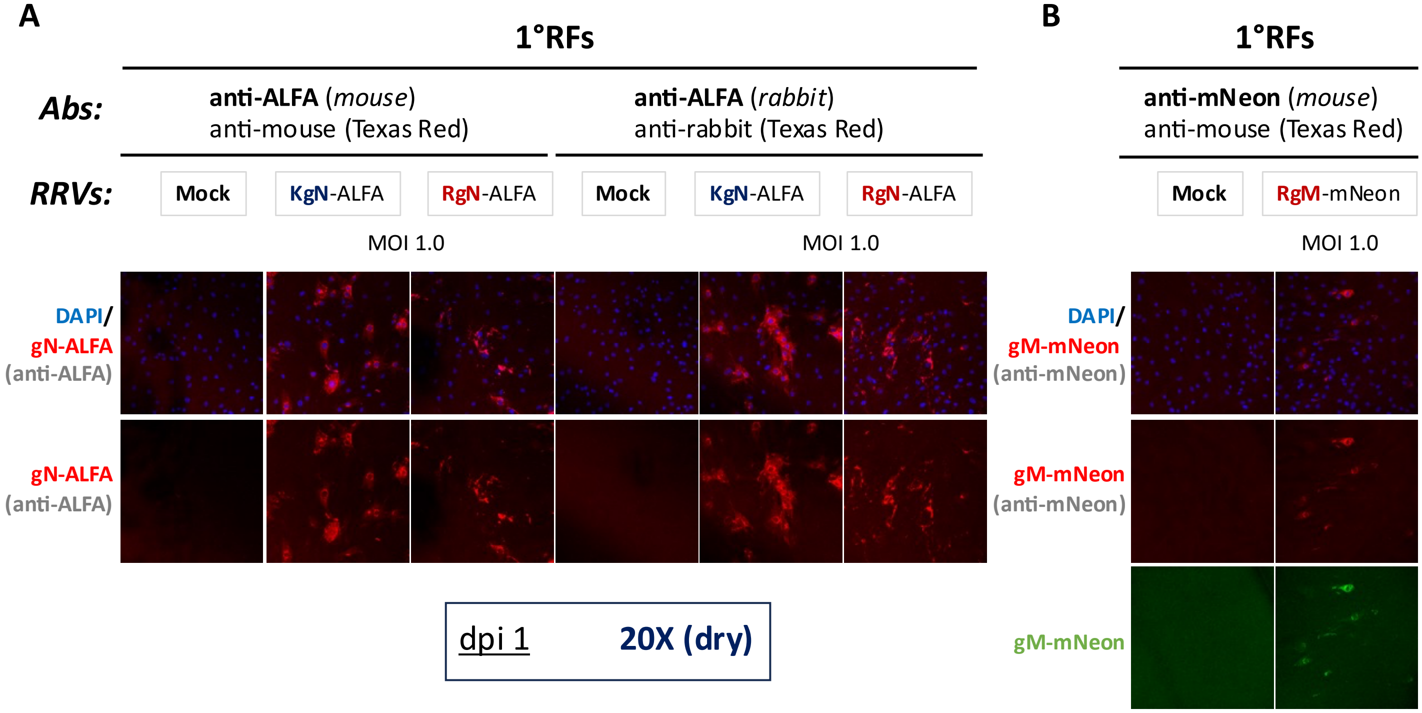

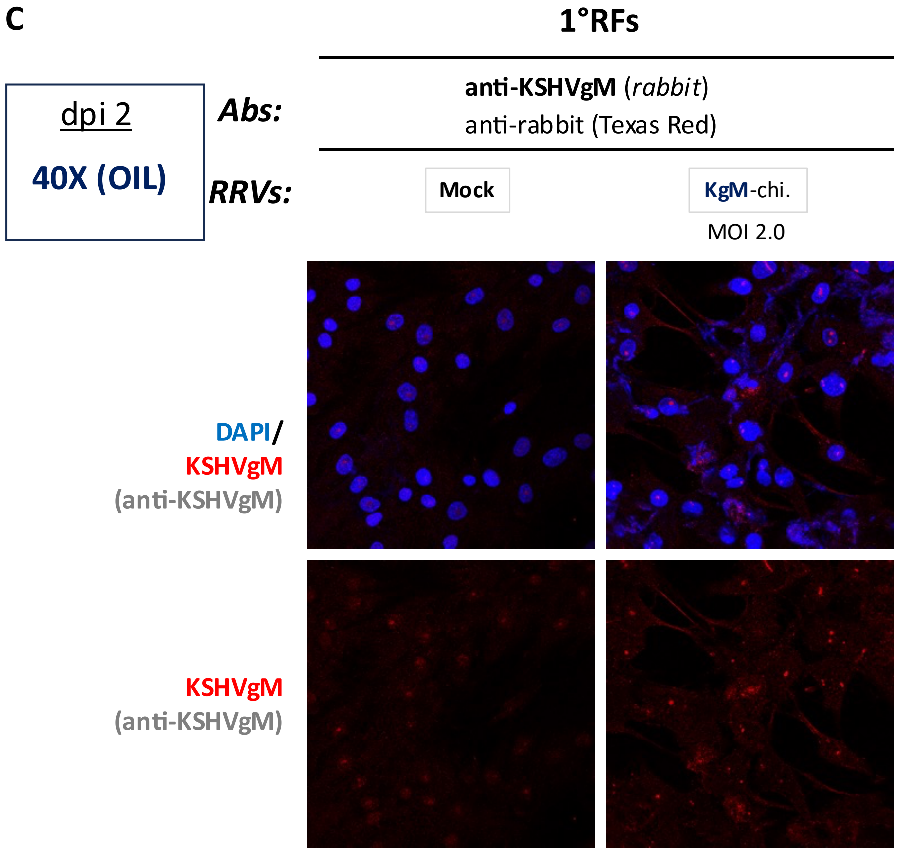

Fig.A7. The antibodies used for PLA recognize their intended targets. A) To validate the anti-ALFA reagents, 1°RFs were infected with KgN-ALFA or RgN-ALFA RRV, or left uninfected, then 1 day post-infection, cells were stained with anti-ALFA (specific for either mouse or rabbit for indirect staining), followed by secondary Ab for Texas Red and counterstained by DAPI. B) To validate the anti-mNeon Abs, 1°RFs were infected by RgM-mNeon RRV, or uninfected, for 1 day, stained for anti-mNeon as primary Ab, stained with secondary Ab and counterstained by DAPI. C) To validate anti-KSHVgM polyclonal rabbit antisera, 1°RFs were infected by KgM-chimeric RRV, or left uninfected, stained with affinity-purified anti-KSHVgM as primary Ab, then stained with secondary Ab and counterstained by DAPI.
